# Supplementary material for: Investment case approach for equitable access to maternal neonatal and child health services: Stakeholders’ perspective in Nepal
Source: PLoS One. 2021 Oct 5;16(10):e0255231. doi: 10.1371/journal.pone.0255231 (PMC8491871; doi:10.1371/journal.pone.0255231)
Supplement: S1 Raw data — (DOCX) [file pone.0255231.s001.docx]

**DIC Qualitative**

**Tool No. 1 Key Informant Interview (KII) Interview with District Public Health Officer**

| **SN** | **Questions** | **Terai** | | **Hill** | | **Himalayan** | |
| --- | --- | --- | --- | --- | --- | --- | --- |
|  |  | **DIC** | **Non- DIC** | **DIC** | **Non- DIC** | **DIC** | **Non- DIC** |
|  |  | **Parsa** | **Sarlahi** | **Baitadi** | **Sindhuli** | **Bajhang** | **Darchula** |
|  | **How many years have you been working in this district?** | I have working for four month in Parsa District. | I have been working about 2 years in a district. | I was here in Bhadra 10 2068. It is around 5 year. | It is complete 2 years. | It has been 18 months. | I have been working for 1 year in the Darchula district. |
|  | **What type of improvement has been currently made in planning process? Can you tell about 14 steps of planning process?** | In Planning process some are improvement in previous year but in spite, now some improvement is needed. In DDC 14 step planning, DDC are conducting every year regularly. In this planning process meeting health related problem does not create broadly and not share to every person such as every health post incharge, every VDC secretory and local community people. In this district, every health worker is not priority for health related information. We talk 14 step of planning process, DDC provide ceiling budget and give plan from VDC then go to district level DDC. District level go to VDC council and VDC council through planning commission then they are passed in regional to central level then apply to district. | Yes, first of all DDC inform to all district stake holder focal person. And Organize meeting at DDC about planning process then evaluate last year program activities. In that meeting, if new matter is needed to include but no any new information in physical years then previous year planning process are continuing, and that meeting interaction with different health related program, etc. broadly interaction to each and every person and at last, planning process pass from VDC council and planning process is done. | In shortcut I can say what should have increase is increasing and what should have decrease is decreasing.  Tell us about the 14 step planning.  It starts from VDC level, ward citizen forum, citizen awareness committee, and then it passes from village council to Illaka council which is followed by district council where the needs and requirements of certain ward or VDC in health sector, education sector, and agriculture sector are passed. Looking at ward level meeting conducted this year and demands in district council, the major demand are in health sector. In health sector also it is mostly about the birthing center. Though we can’t fulfill all the needs, we try to fulfill the major needs based on the population and ecology. | At present top up planning is done in health sector. First of all 14 step planning start at the VDC level which is followed by VDC council where related views and ideas of community people are included. VDC council is followed by Illaka level meeting which includes additional plan as well. Those plans which was developed after district council is submitted to central level. In such a way planning process is completed | In DDC 14 step planning, DDC are conducting every year regularly. | You know that the district was unsuccessful in the MNCH program. The planning process take place but it's not effective.  The planning is made from the ward level which is passed to the central level through VDC council and District council. The plans are approved by the council. |
|  | **With which organization do you coordinate to work in health sector?** | In health sector, all government and stake holders are coordinate with to work in DHO i.e., health, nutrition, sanitation, agriculture, education, women and children welfare sectors.  Health related NGOs and INGOs such as UNICEF, WHO, USAID, family planning and Bidan and Karuna etc. | We don't go to coordinate; they are coming to us to coordinate. We coordinate animal husbandry, agriculture, education and women and child sector etc. We coordinate to them regularly.  **Qn. Can you tell some of the NGO/INGO's name working in the district in close coordination with DHO?**  - Only PSI, plan Nepal, USAID and IPASS working in this district. | Talking about donor organization, UNFPA, UNICEF, Save the Children, GIZ are supporting us. In local level there is not much coordination of NGOs. When required we coordinate with DDC, in education with education sector, likewise in agriculture, sanitation, women welfare office etc. While talking about management we coordinate with district management council. We coordinate with all the NGOs and INGOs as per requirement. | We coordinate with organization working for MNCH, nutrition, HFOMC management and family planning sector.  With which organization DHO coordinate for the budget management?  Though organization don’t support financially, they support technically like. Different organization like NEHAW, Save the children, Care Nepal support in construction and repairing of birthing center, necessary equipment for birthing center, providing training of SBA for nursing staff, CBIMCI for FCHV and health worker. VDC supports for temporary human **resource.** | For the planning process especially DDC, VDC, all health facilities, political parties, including the entire stakeholder for (health, nutrition, sanitation, agriculture, education, women and children welfare sectors gather together for planning. First planning is done at VDC level from where it reaches to Illaka level then to the district level and from district council all stakeholder organization discuss and pass the plan. | They are based on the programs. We coordinate with agriculture sector, animal husbandry, women and children welfare, police and security officer. There is also coordination with NGOs such as CRDS, Suaahara. Almost there is coordination with all the government offices and NGOs depending upon the program. |
|  | **What sort of health related improvement have you observed in this area?** | It is difficulties to say about the improvement in health sector. It's been only 4 month that I am working here, here is only improvement seen in management committee. Another is, some improvement has been in field coordination.  availability of human resource for health, regularity of the services, SBA training, equipment and materials, growth monitoring, According to data, I have not seen but program are conduct continuous. Slightly improvements in immunization sector. Institutional delivery is also increased this year  VDCs are not declared as fully immunized but other 4 to 5 VDCs are in the line of the declaration of fully immunization in fiscal years. | In this district not so much improvement because Madhesh revolution has affected badly. Budget has been deducted due to revolution. Earthquake affected as well. Only one NGO are working in family planning program. Some improvement such as, family planning and MNCH. Only sustaining nothing more than that. | The health ministry conducts district review, then sector review, then national review. In recently conducted review in 75 districts, Baitadi came in number four. In this review 35 indicator are observed. Looking at this we can say there has been development in all sector. In MNCH sector there has been a lot of improvement. The number of ANC checkup has increased, not only 1^st^ but 3^rd^ and 4^th^ visits are given priority, timely iron capsule taking, immunizing with TT vaccine and concept of institutional delivery has increased. In 2068 there was just 7% institutional delivery but it has increased to 71% now. Because of support of donar organization like UNICEF, UNFPA and others, the health workers are given different training like SBA training, they helped in import of equipment's and materials, PNTCT, infection prevention. Because of this Baitadi has reached to number four. | Awareness has increased, home delivery has decreased. Improved nutritional status. Previously growth monitoring couldn’t capture all the children of under 5. Most of the FCHV were not trained for nutrition assessment. All malnourished case was not diagnosed but at present the scenario has changed. At present more cases are detected compared to past but still many cases missing. | After DIC there has been a lot of improvement. ODF declaration was held. This district was declared the fourteenth fully immunized district. Rate of malnutrition is decreasing. There was only 42% institutional delivery before 4 years but now it has reached 71% now in 2072/2073. In sector of safe motherhood pregnant assembly, pregnant rewarding program, invitation card system for institutional delivery and different programs are conducted as mission. Family planning service is integrated with EPI to increase the access. | The health improvement can be measured by improvement in the health indicator. There has been improvement in many indicator compared to previous days. They are: the growth monitoring is above 100%, institutional delivery is also increasing but we are lacking in FP. Our CPR rate is low, this may be due to out-migration. The HIV cases are also being identified. There are testing labs now. The TB new cases finding is also above 100%. The awareness program are also ongoing in the district. |
|  | **What are the reasons behind this improvement?** | The main reason is that improvement to help from district level health related focal person and other is line agency. Political support is the other factor that has helped to achieve these improvements and continuous monitoring has also helped in the improvement. I am working only 4 month in this district so these are the improvement. | The main reason is increasing in the awareness level of the people, service is good, FCHV to provide the information about the importance of immunization, nutrition, ANC checkup to community people.  Another reason is near Birgung National Medical college, different private hospital and governmental hospital has been working in this district so change in the behavior of the people. | Before I came here the number of health workers was less than hundred. More than half of the posts were empty. There is Sarmali VDC, where there was single upgraded ANM who had to cover all the VDC alone, it is biggest VDC of Baitadi with highest population in district. Just after one month of my posting here, there was outbreak of measles. While investigating how the outbreak occurred (WHO was involved in investigation), it was found that 222 children were infected with measles. Two of them died and among 222 children only 4 of them had received vaccine against it. The immunization, the most important task was a total mess back then; this was because there was only one upgraded ANM and she had to look after health post, she had to go give vaccination to children of all wards and she couldn’t do it. It is hard to immunize child due to scarcity of cold chain. Now in Baitadi, all the sanction post is fulfilled. We need man, money, and material for all the work. I have visited all 68 VDC 2-3 times. In some I have visited 6 times also. In some ward there is requirement of two birthing center looking at ecology and population. We are the first district to provide safe delivery service out of birthing center. Women with labor pain of 1 day, 1 and half day, 10 hours can’t climb and go down a hill. So, our practice is to establish 160 delivery sites. We have reached around 110 and we are increasing 4 more. This year we had thought to reach 160 but no matter how hard we worked we couldn’t reach it. At the end of Ashar we will reach around 115. | There are lots of reason for improvement. Support of different organization. Continuous effort and team work is the main things for achievement success. Many training programs are being conducted  Increased awareness Expansion of birthing center. | Awareness among people, governmental services increased and different organizations working in the district. | The health improvement can be measured by improvement in the health indicator. There has been improvement in many indicator compared to previous days. They are: the growth monitoring is above 100%, institutional delivery is also increasing but we are lacking in FP. Our CPR rate is low, this may be due to out-migration. The HIV cases are also being identified. There are testing labs now. The TB new cases finding is also above 100%. The awareness program are also ongoing in the district. |
|  | **What are the improvements in Maternal and Child health (MCH) sector?** | In maternal and child health sector, we watch the data then talking about the improvement. I am only 4 month working here so I don't know detail. | It has some improvement in maternal and child health such as decrease in immunization, decrease in the severity of pneumonia, decrease in maternal and neonatal death etc. you can see indicator then already find out what type of improvement in maternal and child health. | In the sector of child health, this district is 9^th^ district which is fully immunized. There was shortage of vaccine in middle of year so, at that time some child may have missed it. But now we have all vaccines and all children are immunized. In past there was practice of children being fully vaccinated and mother not being vaccinated with tetanus vaccine but now around 80% pregnant women have gotten vaccine thinking this is also for child. The reports of blood test, ANC checkup, operations are being kept safe and they bring it while having delivery. This is positive part of counseling given by FCHV and health workers. At past people of this place didn’t touch their wife when she got pregnant. The religious belief of people was very difficult to deal with. Then we started campaign against it and didn’t let people who didn’t touch their children prohibited in health facility surrounding. Now males carry their baby willingly or unwillingly. This is also a positive change. | Decreased maternal and child death.  Increased institutional delivery.  Increased immunization.  Decreased diarrhea and pneumonia. | Decreased mortality and morbidity related to child health, immunization increased, diarrhea, pneumonia decreased. ANC, PNC and institutional delivery has increased. | As I have already mentioned above that the growth monitoring of the children are increasing, the undernourished case finding is very low. The rural ultrasound program is ongoing in the district due to which the ANC and PNC is increasing. The institutional delivery is increased but we haven't achieved the target yet. Since our CPR is low, NGO and INGO are also working in it. |
|  | **What can be done for additional improvement?** | Talking about additional improvement, in this Parsa District lack of human resources, lack of trained health worker, and lack of building. For example, taking about UNICEF, we have been closely contact about 3 years then a lot of program has been conducted with those organization but not improvement budget. Another next example is UNICEF had been conduct need assessment in 21 birthing center but logistic provide to only 5 birthing center. What benefit did we get from the need assessment done in 21 birthing center, we got nothing. Therefore, the program should also be implemented base on the need assessment done. | Mainly lack of budget. We have no program. Government should be providing a lot of budget in this district then already improvement all sector. | Service center should be increased, health workers should be increased. The sanction post of government is limited, so they can’t increase the number. There was provision of keeping 40 ANM but we have kept 72 ANM. For this VDC supports with finance as well as we also support it. For further improvement the ANM should be kept in community level. In each ward one ANM is required. In Dadeldhura there was provision of three ANM in one VDC. Looking at ecology here in some ward there should be 2 ANM and in some 1 ANM is enough. If we get 310 more manpower we will create example health district. | Fulfilling all the sanction post. Timely supplying of medicine and equipment. Providing training to health staff  Increasing SBA number.  Expanding birthing center, Providing orientation to all the mothers group. |  | The NGO/INGO is few in the Darchula district. We are in category "A", the health indicator achievements are also good. There are about 40-50 NGO/INGOs in our neighboring district (Baitadi) while here is only 2, 3 NGOs and also not so effective. Thus, if we get the support from the NGO/INGOs, then our indicator can be improved faster. |
|  | **What are the problems you are facing in MCH sector?** | The main problem is facing in MNCH sector, lack of staff, lack of staff quarter, and lack of budget. Nepal government provide to staff but staff are not working properly because lack of staff quarter, medhes revolution. So, staffs are transfer to other district. Therefore, a lot of problem in Parsa district. Others are child marriage, teenage pregnancy | We have no new program in this district, lack of nursing staff, and lack of ambulance and also, NGOs are not working properly in this distri,ct. | The area is so remote and big. There is no road in even in 10% of area. Of the rain starts the road gets blocked until Mangsir. The three major reasons for maternal death rate are distance between residential area and health facility, lack of transportation, delay in service. Our goal is reducing delay in service but we still have problem of distance and transportation. Many people while coming to health post have delivery in the way and return back from there. If we get noticed we ask them to visit health facility as we have to do third step management. | Lack of staff and equipment is the main problems of Sinduli. Limited facility of transportation is the next problem. | frequent transfer of the staffs, chhaupadi and traditional beliefs | There is geographical difficultly in the district. If we work for making institutional delivery above 60% then, it would not work due to the difficult terrain of the district. The Birthing center is also not expanded. There was 26 BC in 2070 which is same till now. The advocacy from the local level or DDC can help in making the building for it. The DHO is the software part while the DDC are the hardware. If they make the physical infrastructure then, we can expand the BC which will bring further improvement in the MCH sector. |
|  | **Do you know about District Case Investment (DIC) program?** | I have heard about it in Rauthat District. | I don't know about DIC program. | Yes, I know about it. | No, I do not know about it. | Previously I used to work at PHCC where I got opportunity to involve in UNICEF supported program and DIC is one of them. | I don't know. |
|  | **What are the differences that you have noticed before and after implementation of DIC?** | The previous data has shown the progress and improvement in various fields to some extent. Improvements have been noticed in expansion of birthing center, production of trained health manpower, increase in the number of ANC visits which is the positive aspect to be considered. |  | I already told you when I had joined here, there was just 7% institutional delivery and out of 222 children with measles only 4 were vaccinated. First time we started appreciative inquiry where VDC chief to health worker, politicians, journalists and UNICEF provided support in presence of Nar Bahadur Karki. Before DIC, the budget separated for health by all 68 VDC was just Rs. 5 million (US $ 4500). Currently, the amount separated for health programs by these VDCs has increased to Rs. 1 crore 35 million (US $ 120,000). It may come or not but it is written in papers. |  | Decreased mortality and morbidity related to child health, immunization increased, diarrhea, pneumonia decreased. ANC, PNC and institutional delivery has increased.  Proper planning and budgeting by the stakeholders and their involvement enthusiastically. |  |
|  | **Do you think that DIC should be continued to bring improvement in MCH sector? Why?** | Yes. It should not be limited in planning policy of district but it should reach to mothers group and FCHVs of ward level to make it effective. It should not be only limited to paper works but should be implemented in real scenario in the field and if not done is of no use. So, this should be implemented in our behaviors to aware about the maternal and child health. |  | Yes, it should continue. If possible it should be conducted in 6-6 months if not it should be conducted once a year. It is because the person working may be changed and new comer may not know about it. So, it should be conducted once a year. |  | Yes it is very important to continue. From DIC review workshop it is clear that different stake holder, political party, media, nongovernmental organization, governmental organization plays vital role.DIC is the program which provoke all the related bodies and inspire them. It should be organized every year. |  |
|  | **Now last question, Do you have any additional suggestions?** | For Parsa District, Nepal government should be provide staff and their staff quarter, a lot of budget should be provide, a lot of trained health worker should be provide. And UNICEF should be provided different type's health related program in time. Thank you. | Government should be provided a lot of budget in this district. Budget is not giving in Madhes due to revolution is continued what happen; nothing there is lack of nursing staff and infrastructure is not well. Finally patient have go India (Raksaul) for operation and further checkup. All budget are relies to hill regions. | It will be better if you ask suggestion with beneficiary. The suggestion should come from the community | No, I do not have any. | DIC should be organized every year. It would be better if appreciative inquiry was integrated with DIC review workshop.  Proper monitoring and supervision needed.  It is a good approach for using data, analyzing data, and using the data for proper planning. However, it needs a lot of resources to operate it. It is somewhat complicated and hard to understand different level of stakeholders | No |

**DIC Qualitative**

**Tool No. 1 Key Informant Interview (KII) Interview with Health Worker**

| **SN** | **Questions** | **Terai** | | **Hill** | | **Himalayan** | |
| --- | --- | --- | --- | --- | --- | --- | --- |
|  |  | **DIC** | **Non- DIC** | **DIC** | **Non- DIC** | **DIC** | **Non- DIC** |
|  |  | **Parsa** | **Sarlahi** | **Baitadi** | **Sindhuli** | **Bajhang** | **Darchula** |
|  | **How many years have you been working in this district?** | I have been working about 6 years in this district. | I have been working about 15 years in this district. | Since 4 years am working in health sector where as from 6 month I am working as incharge (dastharthchan )of this facility | Since 2 years am working here at Sinduli. | It has been 12 years that I have been working in Health sector. I have worked for 1 year in  Accham and in this district it has been 11 years complete. | I have been working for 20 years in the Darchula district. |
|  | **What type of improvement has been currently made in planning process? Can you tell about 14 steps of planning process?** | I am not involving in planning process. So, I don't know. But in my opinion planning process start form lower level then go to higher level. | At first start on the basis of data collection then all district stake holders collect data according to population based then conduct meeting. I don't know about planning process in this district.  **Qn.** **Do you inform the approved plan to VDC?**  **-**No | I don’t have knowledge on it.    **Qn. What type of improvement have you noticed in the field of plan and policy?**  The planning is going through positive phase. Because of this positive thinking, we have declared this VDF as ODF VDC. We are declaring this VDC as fully immunized VDC. There has been launching of many different programs like of Vitamin A, Japanese encephalitis, elephantiasis and many other drugs and we are only two people working here in Health post it has become delayed. VDC has also provided budget (40,000) to declare fully immunized VDC. There has been support to buy emergency lifesaving drugs for pregnant women as due to lack of it the life of pregnant women may get in risk.  **Qn. How is the plan and policy making carried out?**  We must start the planning from low level and targeted population and group. To check whether the plan is made or not, we have formed investigation committee. The investigation committee gives nirukul whether the plan is in process or not. We give small plans on dalit, women but are completed rapidly. On making the big plans there may be loss if the plan gets cancelled or we can complete the plan or there is lack of budget. So we make plans that can be tolerated (byoharna sakne) by the committee. We have to take positive thought and also encourage people to have positive thought. To encourage people we can conduct training, meeting, and seminar. Just taking positive thinking won’t help we have to work in positive way too. | We don’t bother much about annual planning. Budget from central level is with certain modification and we accordingly adjust our past year plan…. Of course, we look at health indicators and what target was given by the central level and adjust our plan accordingly. But most of the time our activities are guided by central level. Even if we send our plan and budget, most of the activities and budget heading are removed from there  I do not know about 14 step planning. | Now using our plan and policy by visiting in community, finding out what are the minimum requirement and identifying the problems. The problems are solved on the basis of importance. Solvable problems are selected. On the basis of importance, the problems are taken for workouts and research and follow-ups are carried out in cross checking to find out what is our goal and why did we chose that plan to achieve the goal, the we try to reach that goal.  **Qn. Can you explain us in brief how our plan and policy works in VDC level, district level or in this regional level?**  In district level, we have discussion and co-ordination with district’s responsible person and accordingly we are also participated in discussion and meeting of district level. Similarly, NGO/INGO also help in that. Accordingly it is done by co-ordination and discussion of all responsible persons.  In VDC level, before selecting any plan in ward level there is formation of ward citizen group **(wada nagarik manch)** and one coordinator is selected from ward citizen group and president of mother group, female health workers (FCHV), health associated clubs, NGO/INGO facilitator friends and political parties and by conducting meeting among them and by discussing among themselves to select necessary plans, plans according to problems, everybody has key role in it. Public awareness also falls in it. In VDC there is peace committee which also falls in it. Everybody has role/participation in this selection. The plan is selected by special represented from women, dalit, disable.  It has been 12 years that you have been working in health sector. How are you working in health sector by coordinating with NGO/INGO? | The planning is started from the lower level. The VHW and upgraded ANM make plans of the wards. They plan when to conduct ORC clinic and its places. They also come up with some other health services that the village people needs. They also meet with the FCHV and record the pregnant women of the wards. Then, based on that, we make health planning for the district. After that the district makes the plan based on the VDC's plan. We always make plan in the Shrawan month and works for whole year. |
|  | **With which organization do you coordinate to work in health sector?** | First of all coordination is done with governmental such as DDC, Education office, agriculture, women welfare etc. and another is non- governmental office such as family planning division, Plan Nepal, UNICEF and USAID. | I don't know  **Qn. Can you tell some of the NGO/INGO's name working in the district in close coordination with DHO?**  **-**Only Plan Nepal but I am not conforming | We are coordinating with VDC, DHO, women welfare, education, NGO / INGO | We are coordinating with VDC and different non-governmental and UN organization. | Nepal government is solving problems with the help of different donar organization by receiving financial help and manpower. Our need/requirement from donar organization is listed and one minuting formed by grouping HFOMC with us is recommended, then it is recommended from DHO etc. If DDC’s recommendation is necessary then by recommending, what are our problems? For example; if any equipment is for birthing centre is needed then minuting is done from VDC, management committee and then recommendation from DHO. Here we have UNFPA, UNICEF. Mostly UNICEF has been helping us in our birthing centre. If we ask donar organization following rules and procedure then they do help us.  While declaring this Bhajhang district as fully vaccinated VDC, UNICEF, UNFPA had played great role. They helped us to solve financial problem as well as during staffing. | There is no organization that is directly working in the Darchula district. Suaahara is working in nutrition; Pahal Nepal is working for sanitation etc. |
|  | **What sort of health related improvement have you observed in this area?** | Some improvement then previous year such as; increased in vaccine coverage. In nutrition sector, some improvement then previous year due to Bal Vita Program. Another improvement reason is staff has got a lot of training and information about health. ANC is also improvement. Increased in institution delivery. | Some improvement then previous years such as child health, Immunization, sanitation, nutrition sector and family planning is also improving. | Increase in ANC and PNC checkup. Previously people didn’t visit here now they visit here. In immunization it is 100%.  *Previously there were only three staff but now it has increased, because of which we are able to go in field for growth monitoring.*  Regularity of the services, SBA training, equipment and materials, growth monitoring, Prevention of Mother to Child Transmission | There are lots of improvement in Sinduli. ANC, PNC, institutional delivery has increased.  Child are fully immunized. Improvement in nutritional status of child.  **Qn. What type of improvement have you noticed in sector of MNCH?**  I have already told some of them.  Different organizations, VDC are supporting us. Decreased in infant health rate. | In this mostly P1 programs has been given priority. We provide primary health care service rather than curative service. It is better to prevent our self rather than being diseased.  *”When a child is born in the community, we provide the child’s* ***naisargik adhikar (child right)*** *like vaccinating the baby with all the available vaccines or vaccination is done without leaving any child. we have formed greeting/welcome cards for pregnant women. In every ward we go through list of pregnant women and make them compulsory for four times checkup, to get vaccinated with TT, to take albendazole, iron tablets, to take green leafy vegetable in one of the meal”*.  We have formed Institutional Delivery Support group to declare Lamatola health post as “no home delivery VDC.” Likewise, in UNICEF there is formation of watch group but it is not commenced in our VDC. We have been running Institutional Delivery Support group by participating the youths. We have provided stretcher in all wards to carry pregnant women to the health post. Similarly, we have formed greeting/welcome cards for pregnant women. We have created pregnancy calendar. In every ward we go through list of pregnant women and make them compulsory for four times checkup, to get vaccinated with TT, to take albendazole, iron tablets, to take green leafy vegetable in one of the meal. We have slogan, “everyday four foods” (harek baar khana char) We have been providing PMTCT service for woman during breastfeeding, in addition full breastfeeding, additional food for child, then vaccination, likewise we also talk about family planning. | Health improvement can't be seen at once, it’s a long term process. The little improvement are seen in the immunization where number of the immunized children is increased, the number of the growth monitoring children are also increasing each year and the diarrheal, Dysentery and Pneumonia diseases are decreasing. In case of maternal health, ANC and PNC is increasing each year, the ANC visit is also as per protocol. |
|  | **What are the reasons behind this improvement?** | The main reason is the Increasing in the awareness. In my opinion, It is a government policy. More people are literate. Access of health worker in every health post. | The main reason is the Increasing in the awareness level of the community people. FCHV to provide some Information about the need for proper nutrition during pregnancy and lactation through FCHVs. And another reason is, mother’s group meetings conduct regularly by FCHV and also health worker are providing information about ANC check-ups. | Because of availability of manpower, due to good support of school and due to support of community. | Fulfillment of sanction post. Increase in awareness, team work is some of the reason for it.  Due to awareness, broadcasting of information from media. We are conducting awareness program and training. It has contributed a lot to improve the health status. | Nowadays in most society health empowerment, different programs, meeting, seminars have been encouraging people in some way. Two years ago, District Investment meeting was held. In that meeting one sir, I forgot his name; he washed negative thinking and made people think positively. He changed negative thinking of many friends and made them think positively. I am also moved by his way. I could declare first vaccinated in fast manner. If we take leadership and move forward with positive thought, even inactive friends become active.  By having feeling that, since we are born to be youth we have to do something, now that positive thought of our health worker has leaded our Bhajhang district in ninth rank.  Looking at the child health, the major issue is vaccination. In Bhajhang district every child has got vaccinated. Everyone is doing sustainability of vaccination. In case of maternal health, our all health post /organization has been turned into birthing centre except for three health post. MDGP doctors also available from NSI because of which in critical condition, CS is done in Bhajhang district. Another side of NSI is that, it has provided MLP training to paramedics and made them capable. MLP training has updated us because of which we can now diagnose different new-new types of disease. The maternal death rate has also been zero from six month. This is also possible because of birthing centre and safe motherhood service. | There are many reasons behind these improvements. One of the main reason is awareness in the people without knowledge, nothing can be changed. The improvement in the ANC and institutional delivery is due to the incentives by the government and the commitment of our ANM and FCHV to bring them in health facilities. They are also active because if the institutional delivery is not high then, it can hamper in their jobs too.  In immunization and ORC clinic sector, service provider and organization should established the ORC clinic in the remote areas.  The organization is also working on nutrition. The employment opportunity is also increasing which also help to make improvements. |
|  | **What are the improvements in Maternal and Child health (MCH) sector?** | Talking about maternal and child health some improvement is ANC checkup. Expansion of birthing center. Proper supply of medicine for community people such as TT, Albendazole and a lot of medicine. In child sector, some improvement is nutrition area due to Bal Vita Program. | Talking about delivery, previous home deliver conducted at home but now a days institutional delivery has increasing, child suffering from any disease such as diarrhea then come to health post for checking. The iron intake, vitamin supplements has increased too. |  | I have already told some of them.  Different organization, VDC are supporting us. Decreased in infant health rate. | For further improvement, which is in community level, friends working in community are new and energetic. They bring new and innovative ideas. All friends think we have to follow him. The leader of district also has positive vibes. Our doctors are also energetic. Friends bring new innovative plans. Time to time our work should also be reviewed. We have to be aware of which position we are in and what should we do. If we make plans to reach our goal we will move towards improvement. | Ans in ques 5  **Qn. What about nutrition and breastfeeding section?**  -Hand washing is improved significantly since every program has now incorporated the sanitation, personal hygiene and WASH part.  The nutrition sector has been also improved. The malnourished is very low compared to previous days. |
|  | **What can be done for additional improvement?** | Staff training will be providing regular basis, time to time. National level has made big planning, rule and regulation. That rule and regulation know about all community people and back ward community people. | Broadly advertisement about health and education, advertisement in uses of family planning services, advertisement about institutional delivery also nutrition for child. First of all awareness create for sanitation and hygiene maintain in local, Dalit, Janjati community people. In Terai area, most of people are made toilet in home but they do not utilized regularly because no awareness. | Maintaining sanitation, behavior change is the most important part. Team work, increasing awareness program. | For improvement we have to move forward, each and every person should move forward to have improvements. We can use local resource like we can use soybean to make lito, sarvottam pitho, also from wheat but we use market sold sarvottam pitho. We are dependent on others, we act like parasite. Because of thinking that, “we can’t do anything” and being dependent on others we can’t move towards improvement. We can ask help of VDC, DDC, and local development ministry for it. | For further improvement, which is in community level, friends working in community are new and energetic. They bring new and innovative ideas. All friends think we have to follow him. The leader of district also has positive vibes. Our doctors are also energetic. Friends bring new innovative plans. Time to time our work should also be reviewed. We have to be aware of which position we are in and what should we do. If we make plans to reach our goal we will move towards improvement. | There are many things to do to make further improvement in MCH sector. The PNC is not increasing, if we also have some incentives or refreshment for PNC to both women and HWs then, they will also show work on it. The main thing to see improvement is awareness. If the people are unknown to things then they will not utilized it. Therefore awareness program should be there. |
|  | **What are the problems you are facing in MCH sector?** | In Birgung, For Patient checkup does not come in governmental hospital. All patient are go to private hospital in Birgung for health checkup, these are the main problem in Birgung**.** | In Terai area first problem awareness, second problem is budget, third problem is nursing staff and also lack of human resources. | Some clients hesitate for checkup. Severe case are delayed in refer. Behavior of some health worker is not good. | We are facing lots of problems in MNCH like maternal death, infant and child death. | When we talk about child health, we have now got the IMCI training. Before when we didn’t had full IMCI training we had difficulty to cure many diseases in integrated form. When there was no birthing centre and delivery was carried out in home, due to lack of hygiene, problem of hypothermia, infection and severe illness was common but now it has been improved. | The BC centers are established but there is no suction machine, electricity and no heater. It's like only for name. The BC is also a kind of Operation Theater, it should be clean and infection free. There are only 3, 4 room for everything. They have to run EPI clinic, HP, ORC clinic, OPD, store and BC too. Therefore it is only established for name. Therefore, a different building for BC with 3 rooms should be constructed or a HP with different section for BC should be established. There must be atleast an ANC/ PNC room, Delivery room and a waiting room. |
|  | **Do you know about District Case Investment (DIC) program?** | I don't know about DIC | No | IC planning process is systematic which was evidence based and not perception based and the plans developed were focused to achieve the set targets in IC districts which was a positive sign of stepping towards evidence-based planning | No, I don’t. | Yes, I was present there in first District Investment case program. We had done some planning during that time. I was present in second review too. We even discussed on plans, if it is fulfilled or not. This group has played important role in leading this health sector forward. | I don't know.  Maybe it is the engagement of external development partners for supporting infrastructure and human resource. |
|  | **What are the differences that you have noticed before and after implementation of DIC?** | Don’t know DIC | Don’t know DIC | Inclusion of the political parties’ representatives, as one of the stakeholders during workshops, was an important factor.  Increase in MNCH indicators | Don’t know DIC | Before starting DIC there was development in health sector, as it is now in two and half years after DIC. People from district have been moving towards leadership but beforehand even we didn’t had feeling that we have to do new work, we have to do innovative works in field of Public health. We only worked according to protocol of HMIS. We were even inactive in works of Public health. After running DIC workshop we got feeling that we have to do new works. We saw in DIC workshop that a person without hands and legs can also work because of his ideas and Sir had presents that, that particular idea ked to better work. It was shown that after we have been born on Earth we have to do something better. Even VDC secretary didn’t give much attention to health. When the village FCHV brought any pregnant women to health post, VDC started giving rupees 200, 500 as encouragement. After DIC workshop VDC secretary has also been helping us. | Don’t know DIC |
|  | **Do you think that DIC should be continued to bring improvement in MCH sector? Why?** |  |  | Yes, it should be because it Makes people believe that it works, then the change is certain”, |  | Yes, it should be continued. If possible our achievement according to our planning should be reviewed in 6-6 months and if DIC continues we will become aware of it. It empowers for our work and it has encouraged us to do some innovative works. So, I think it should be continued. |  |
|  | **Now last question, Do you have any additional suggestions?** | More focused on birthing center. In birthing center, not sufficient equipment, building and another is awareness program must be launched for back ward community people who suffering from different types of disease.  Proper budgeting by the national level to VDC. | More focuses on how to community people are use in your toile and another is nutrition sector. Government should be providing a lot of budget in Terai area to create awareness for community people. | We should increase the number of field worker and FCHV. | I think UNICEF need to be more responsible to provoke the entire stakeholder time and again. All the organization working for health must realize their responsibility to improve the condition of Sinduli.  Government district stake holder must realize their duty and responsibility so that we can improve the health status | Most DIC workshop has gathered many participants from different sectors to find out common problems so, by gathering participants and chiefs from every sector the workshop has been running. For that I am grateful to them and this workshop will lead our health sector forward. The indicators of health will also increase and we hope we will keep receiving help from your side.  Bottom up planning and proper budgeting as explained by DIC. | The ANMs of the BC are of contract basis. If they are from the sanctioned post, then they would retain themselves here but they are always searching new opportunities. They usually come for few days in the HP since they say that they have to prepare for the public service commission exam. Therefore, there full attendance and retention is problem. The building of the HP are made of stone and has only 2, 3 rooms. The roof leaks during rainy season and the mouse eats up all the medicines from the store. Thus, if the buildings of the HP are well constructed, furnished and equipped then, we can provide better services to the community people. |

**DIC Qualitative**

**Tool No. 1 Key Informant Interview (KII) Interview with Public Health Nurse**

| **SN** | **Questions** | **Terai** | | **Hill** | | **Himalayan** | |
| --- | --- | --- | --- | --- | --- | --- | --- |
|  |  | **DIC** | **Non- DIC** | **DIC** | **Non- DIC** | **DIC** | **Non- DIC** |
|  |  | **Parsa** | **Sarlahi** | **Baitadi** | **Sindhuli** | **Bajhang** | **Darchula** |
|  | **How many years have you been working in this district?** | I have been working about 2 years in this district. | I have been working about 4 years in a district. | It has been 14 months. | It has been 20 years. | In this district I am working from 2 years. In public health it has been six month. | I have been working for 1 and half years in the Darchula district. |
|  | **What type of improvement has been currently made in planning process? Can you tell about 14 steps of planning process?** | We have guideline. Program should be conduct according to guideline guide line, all information are given their. Thus, we are flow guideline for conducting health related program. I don't know about 14 step planning.  **Qn. Do you involved in any planning process in this district?**  -I am not involved.  **Qn. Do you coordinate with the DDC while conduct planning process meeting?**  **-**DDC don't coordinate with you. Only involve DHO in this district. | First of all DDC conduct planning meeting and in that meeting, All district stakeholder, program related focal person and FCHV are involving in planning meeting. All Bara district problem are creating in this meeting then problem is find out. According to problem we all are made plan in this district then the plan are pass VDC council. Likewise planning process is done. DHO only involving planning process meeting so, I don't know detail about planning meeting process. | I don’t know much about what was done in the past. Now while making plans first we take suggestion from VDC people about their needs and priorities. While making annual plan we focus on suggestion received, requirement and need. Then by making the annual work plan it is sent to central level.  I don’t know much but according to what I have heard, first it is carried in ward level then it is passed to VDC followed by district level. From district it is carried toward upper level. I don’t know about it in detail. | We make plans here. We conduct meeting of management committee in VDC and DDC. We conduct RACC meeting. PHC level and health post level ma health samandhi management system khada gareko chau. | Mother’s group, FCHV, health post incharge come together and bring subject to discuss on and we have district review meeting time to time then make planning on how to make district move forward. Like if we have review meeting at the end of year, what will be done in the next year is planned. Every field related to health sector, like family planning, safe motherhood is discussed in the planning. | I don't know about the planning process, 14 step planning and I am not involved in the council too. Only DHO sir goes to the DDC and VDC council. |
|  | **With which organization do you coordinate to work in health sector?** | In health sector, all stake holders are coordinate with you. And, we have some private organization such as Birgung National Hospital, Private Hospital, UNICEF, Family Planning, iPAS and another is Lion club coordination with DHO. | From the government section, we have now some sector such as DDC, women and child sector, agriculture office etc. we coordinate regularly.  **Qn. Can you tell some of the NGO/INGO's name working in the district in close coordination with DHO?**  **-**Only PSI and plan Nepal are working in this district. We have coordinated with you for further working. | GIZ, One heart worldwide, HC3, Care Nepal, UNICEF, Aawaj Abhiyan, Etc. Aawaj Abhiyan works for uterine prolapsed. HC3 works for family planning. Similarly one heart worldwide supports for safe motherhood. They have provided equipment’s for birthing center, training like IMCI, ultra sound training (still continuing), provided mobile for health facility but communication charge is managed by them. | We have contact with Care Nepal, SIDS Nepal, UNFPA and many more. | We coordinate mostly with the health related NGO/INGO. Health facility coordinate with VDC secretary and by mobilize youth clubs, mother’s group etc. | There is very few organizations working in the health sector. Only Suaahara is working in the District for health sector. |
|  | **What sort of health related improvement have you observed in this area?** | In Parsa district, some improvement in birthing center. Nutrition status has also improvement then previous year, Increase in ANC checkup. Trained SBA has got training. In terai, The ANM gets transfer before 6 months. Due to Madhes revolution all staff has transfer to other district. In Parsa district, Nepal government should be provided a lot of staff, SBA and ANM. Other improvement is awareness, good services provided by health worker to health post. Budget allocate on time. | Last fiscal year compare to some improvement in fiscal year 071/072. For example increase in awareness, immunization; uses of family planning services, FCHV are creating awareness door to door for ANC and PNC service. Now also treatment for diarrhea cases, Nutrition and some increase in institutional delivery. | UNICEF supported shelter home. Due to shelter home many pregnant women were benefited. Now the shelter home program has phased out. There was even management of hygiene kit for pregnant women    Many training programs are being conducted nowadays. NTAG has also supported us. I forgot to add that NTAG is working to improve nutritional status of pregnant women and malnourished children. | There has been launch of safe motherhood program on funding of USAID, increase in birthing center, increase in visit of pregnant women, availability of CEONC services. Previously working doctor “Bhutel” had started CEONC service at district hospital. There is lack of blood bank so we manage blood by making phone calls. | Like in case of café motherhood, previous year we had 42 birthing centers including BONC, CEONC and this year we have established three birthing centers. We are providing delivery service and safe motherhood rate is increasing. PMTCT services will be initiated after the starting of birthing center.  Providing family planning counseling and device during the time of PNC visit. While talking about the daily nutrition counseling is done.  DIC had been instrumental on making Comprehensive Emergency Obstetrics and Neonatal Care (CEONC) service available and timely utilization of the services in coordination with FCHVS and health facility staffs. | There has been lot of improvement in health sector. Previously, there was many home delivery but now atleast there is 50% institutional delivery. The Suaahara program is also helping in the MCH sector. It focuses on the 1000 days mothers. They have even reach dot each and every woman and had counsel them for check-up due to which there has been increased in ANC and PNC visit too. Thus, the MCH sector has improved compared to years before. |
|  | **What are the reasons behind this improvement?** | This is due to communication, training has conduct on time, VDC level has conducting meeting regular basis. That's the improvement.  **Qn. Next reason behind this improvement.**  **-**don't know. | The reason is the increase in awareness, increasing in education and communication level of the people. Two and four Doctors are every time available in Each and every health post, also FCHV are hard working in this district for community people. | Due to support of NGO/INGO, DHO has been able to improve in health sector. | There was implementation of safe motherhood program in 2064/66 because of which there was increase in awareness among people about institutional delivery; we conducted programs to reduce the rate of maternal deaths. There is increase in awareness about incentive that is received after and during pregnancy period. There are people who still lack services due to ecological barrier. | People are becoming educated with time and they get information from F.M, television, also this means uses are being increasing day by day. Day by day clubs are being developed like Aama samuha.at present number of NGO/INGO has been increased. | The NGO (suaahara) is the one who is working for the mother and children due to which these improvements are seen. If there are other NGOs too, then it will support us more to make further improvements. Together with them, we have also reached to the remote areas and have provided services to them. Thus, we can achieve success if both side work together. |
|  | **What are the improvements in Maternal and Child health (MCH) sector?** | No home delivery, institutional delivery has increase, in child health sector some improvement has immunization. Bal vita program has more support to nutrition program. Good coordination between FCHV and Health post staff. Good health services provide to Birgung private hospital for patients.  Qn. **How many VDCs are decelerations of fully immunization in this district?**  **-**None of the VDCs has been declared as fully immunization in this district. | Talking about maternal and child health sector some improvement for example, increasing in institutional delivery and increasing in uses of family planning services. The iron intake, vitamin supplements has increased too. Thus health of child and mother has been improved.  And also increasing in immunization rate for child health. | Institutional delivery has increased, decreased maternal and child death, decreased still birth, increased awareness about hygiene, nutrition and immunization, continuation of different training, distribution of Ready To Use Therapeutic Food (RUTF). | Previously there was no program of safe motherhood but it is there now.  There has been launch of safe motherhood program on funding of USAID, increase in birthing center, increase in visit of pregnant women, availability of CEONC services. Previously working doctor “Bhutel” had started CEONC service at district hospital. There is lack of blood bank so we manage blood by making phone calls.  What are the reasons for this type of improvement?  There was implementation of safe motherhood program in 2064/66 because of which there was increase in awareness among people about institutional delivery, we conducted programs to reduce the rate of maternal deaths. There is increase in awareness about incentive that is received after and during pregnancy period. There are people who still lack services due to ecological barrier. | Previously there was no service of CS (caesarean section) at bajhang. Now the scenario has changed. With the support of NSI, CS service is available at district hospital of bajhang. MDGP doctors are available. Ectopic pregnacy was handled at district hospital. If we lack those services we need to referrer to dhanghadi.  Awareness has increased.  Immunization has increased.  CHX project was implemented with the support of JSI. CHX prevents infections of cord. | The people are aware about the ANC visit, importance of institutional delivery, danger sign of the pregnancy and delivery, caring of the newborns, Immunization and family planning. Not only the people from here but also the people of the remote areas are aware about these things. |
|  | **What can be done for additional improvement in MCH sector?** | Program should be conduct on time. And another is Nepal government should be provide a lot of staff and their staff quarter also available in this district. We have no staff quarter. For monitoring, a lot of monitoring staff are their need. We have no monitoring staff. Local NGOs and INGOs will be managed staff and their quarter. To be provide awareness through health worker to community people on time. Then, we can see further improvement. | Taking about Bara district lack of budget, lack of building, lack of Nursing staff and trained health worker, lack of human resources these are the main problem of in this district. Thus, if this problem can manage then we can make already progress. | Need identification and addressing it. Due to phase out of temporary staff birthing center lacks nursing staff. It would be better if nursing staff for birthing center are sponsored by different organization. It would be better if UNICEF manage stretcher in all VDCs. | There is lack of manpower here. We have 20 birthing centers but we don’t have Sanction post/darbandi for ANM.  Those who are there they lack SBA training. The sanction post is not fulfilled. We don’t have permanent staff. The staff that gets training take transfer and go away. There is lack of materials in birthing centers. There is lack of delivery sets. Due to lack of awareness pregnant women visit only once for ANC visit. | Establishing new born corner and providing supporting equipment.  Distributing nyano jhola.  Timely demanding and supplying necessary equipment.  Health facility lacks buildings so establishing building with the support of donor agency. | We have to work a lot for improvement. The work based on the situation. Darchula district is remote, geographically challenged and mountainous district. There is lack of transportation in the VDCs due to which we couldn't make much improvement here. If there is transportation facilities in the VDC, fulfilled health manpower in the health facilities then, we can achieve further improvements. |
|  | **What are the problems you are facing in MCH sector?** | The main problem is staff; none of birthing center has not full fill main power. SBA training will be conduct time to time. Minimum 5 SBAs training will be provide by UNICEF. Lack of building and Budget should be allocated on time. | In Bara district, Main problem is budget also lack of Nursing staff and another is lacks of trained health worker are facing in MNCH sector. | High prevalence of malnutrition among the children, poor nutritional status of mother. Lack of awareness, poor referral system, unable to maintain sanitation and hygiene. | There is problem of uterine collapse. We conduct campaign related to it. The campaign is conducted but the problem is not solved due to lack of camp.  The manpower should be available, the materials and equipment needed should be fulfilled, there should be provision of providing training, SBA training should be given to all of the staffs working in birthing center, there should be provision of providing refresher training to ANM, there should be training of IP (infection prevention) | Lack of human resources, buildings materials and equipment.  Due to the geographical barrier pregnant women could not visit health facilities. | The female still backward, they can't make their own decision. The villages are very far from the road way, they have to pass through river and forest to reach the villages. When there is a labor pain, how can they come in the hospital foe delivery? Therefore, the roadways are the main problem here. To improve the health sector, we need coordination from other sector too. |
|  | **Do you know about District Case Investment (DIC) program?** | I have heard about it. DIC means to identify the cause of the problem of VDC level. | Don't know | I know little about it. | No, I don’t. | Yes, I know about DIC after participating in the review meeting. It focuses on requirement of enhanced collaboration and coordination among various stakeholder like -political parties, health worker, VDCs and others to address these issues has made the implementation effective and easier. Otherwise, it is very hard to convince political leaders for investment in in health. | I don't know. |
|  | **What are the differences that you have noticed before and after implementation of DIC?** | As we know that, safe motherhood program has more improvement then previous year. I have no about it detail but some improvement after DIC. We have conducted Need assessment in birthing center. Thus, we have find out some improvement then previous years. Due to DIC, If the meeting had been conduct in the VDC then all people are involved in their meeting. |  | The main objective of DIC project of last year was to declare Baitadi as fully immunized district. It was possible to accomplish due to help of health incharge, health workers and political parties. This year our aim is to congratulate women who had delivery in birthing center. We are going to declare fully institutionalized delivery district within 2074 Ashar, for this we need help of different organization and health workers as in past. To do so we are carrying out the DIC project to motivate them to help us. In this program Nar Bahadur Karki sir had arrived who provided motivation. Politicians and secretary have committed to help us. They have helped us like they have brought more budget compared to past. For accomplishing the goal of constructing 160 birthing center, VDC has separated budget for different purpose like for constructing building, buying equipment, for management of birthing center. Budget has been separated specially for construction of Birthing centers. |  | Previously children were not fully immunized but now this Bajhang district is declared as fully immunized district. It was included in DIC action plan. Establishment of CEONC center, PCNOBO center and Blood bank by coordinating with Red Cross because of which we don’t need to go to Dhangadi to receive blood.  IC had been instrumental on making Comprehensive Emergency Obstetrics and Neonatal Care (CEONC) service available and timely utilization of the services in coordination with FCHVS and health facility staffs. |  |
|  | **Do you think that DIC should be continued to bring improvement in MCH sector? Why?** | The program must be ongoing but it's should be modified. Such as, if we are informed about your arrival (one week ago) we had made full preparation. |  | Yes the DIC should continue. It helps to share information about how much investment is done in certain place and how much development has been done, it helps to know about development of own VDC and other VDC as well, how are they working, how can we coordinate, what should be done is discussed in the group. There is exchange in views, knowledge between each other and people participating are also motivated. So, the DIC should be continued. |  | Yes, it should continue because it helped us to establish 5 CC centers with necessary materials and equipment, we established waiting home with need equipment’s. We now just don’t check rooms for birthing center but we inspect how the New born corner is. It should be held every year if possible if not then it should be held once in three to five years. |  |
|  | **Now last question, Do you have any additional suggestions?** | If we are provided with budget, then it should be handled by only one person. In previous year DIC workshop the budget was handled by more than one person, which made difficult to provide DSA to the staff. It is difficult, if there are 50 participants but we had to provide DSA to more than that person. Also, the budget should not be given in combined form. DIC has been implemented years ago but no monitoring has been done. The monitoring of the program should be focused. | Extra suggestion is given to those from whom we have some expectation it means benefit. If we don't have any benefit why should we suggest. | No, I don’t. | The sanction post of ANM should be fulfilled. The infrastructure, material and equipment's should be provided; there should be provision of SBA training and refresher training. | No, but while the DIC meeting was held I had no much concept about it so, I think there should be some training programs.  Monitoring and supervision should be on regular basis | We have very strong motivation to work and make improvement in the mother and child health. There are not many organizations in this district. We don't have support from other organization. The HW are also not enough here. The government hasn't fulfilled the sanctioned post of upgraded HP or PHCC.  Therefore, I would ask for the external support from the different governmental and non-governmental organization. |

**DIC Qualitative**

**Tool No. 2 Key Informant Interview (KII) Interview with Local Development Officer**

| **SN** | **Questions** | **Terai** | | **Hill** | | **Himalayan** | |
| --- | --- | --- | --- | --- | --- | --- | --- |
|  |  | **DIC** | **Non- DIC** | **DIC** | **Non- DIC** | **DIC** | **Non- DIC** |
|  |  | **Parsa** | **Sarlahi**  **(Planning Officer)** | **Baitadi** | **Sindhuli** | **Bajhang** | **Darchula** |
|  | **How many years have you been working in this district?** | I have working since 7 month in this district. | I have been working past 14 years in a district. | I am working here from 9 months | I have been working 1 year in a district. | I have worked here from six month. I have worked form 2062-2066 in different positions sometimes as planning officer, sometimes as acting officer of municipality, project coordinator. | I have been working here for 11 months. |
|  | **What type of improvement has been currently made in planning process?** | In DDC 14 step planning, DDC are conducting every year regularly. In this planning process meeting,  We talk 14 step of planning process, DDC provide ceiling budget and give plan from VDC then go to district level DDC. District level go to VDC council and VDC council through planning commission then they are passed in regional to central level then apply to district. | Yes, first of all DDC inform to all district stake holder focal person. And Organize meeting at DDC about planning process then evaluate last year program activities. The planning starts from ward level to VDC level and to Ilaka level which goes to District level and finally plan is approved. | We take it by passing the 14 steps and then implement it. In my effort from the VDC council, different departments are given the role to lead the local areas and have made policy which will be in continued matter. Another policy made includes NGO/INGO and different organizations were they gather and make decision that the related different organization head will lead and plans are made transparent, discussed. The grass roots are raised from residence, village to district, urban and programs run and end by completing the 14 steps. | The planning process is based in 9 steps**.** The plans are made in the ward level from the focal person of wards. The plans are discussed in the ward citizen forum with the involvement of people from different sector. Likewise, the plans from the nine wards are passed to the VDC level. Again, the plans are discussed in the VDC council. In VDC also, the people from different sector such as, women and child, agriculture, are involved in the planning process. Those plans that can be carried by the VDC are approved by them while the other are again submitted to the district level.  In the district level, the plans from all VDCs are compiled, discussed and prioritized the plans in a council. The different programs have their own budget ceiling under which we have to allocate the budget. We also review the previous plans and prioritize the plans for coming year. In case of disaster, the planning is again set different at that time of urgency. Sometimes, the plans that we have sent is not what we get from the upper level. | About 14 steps planning, governmental –nongovernmental organization should continuously monitor after the process after the completion of workshop. The planning process reached to the district council after completing all processes  **Qn. Tell we about it from ward level to district level in systematic planning process.**  First it starts from local level then it proceeds to ward level. From there it proceeds to VDC, then to VDC council and then passes to Illaka. After Illaka meeting, we sit for sectorial committee. Then it moves to integrated committee then we move to DDC meeting and then DDC council and then plans are brought to central level. | There has been improvement. The planning is done from different sector and committee. Some are done from the regional sector too. The planning is not successful sometime due to the many reasons.  **Qn. Can you tell about 14 steps of planning process?**  -The planning goes through different council and ends at the central level. The plans are approved only when they are passed by the councils. |
|  | **How do you coordinate in health sector? (Budget)** | In health sector, all government office and other stake holders are coordinate with to work in DHO. Health related NGOs and INGOs such as UNICEF, WHO, USAID, family planning and other health related coordinate budget then go for further apply for the district. | They are coming to meeting and to coordinate. We coordinate animal husbandry, agriculture, education and women and child sector and DHO etc. We coordinate to them regularly. Only PSI, plan Nepal, USAID and IPASS working in this district. | Health sector is based on the umbrella concept. While running the health related programs, health sector is given leading role. Policies arising from the health sector are passed from district council and are addressed in the group district policy. The interested donar organization by coordinating and discussing with health sector, they are influenced to invest in the place where the need is high. | We coordinate with them when we have similar programs, when our objectives are same. Also, different health organization coordinates with us for the support. Likewise, we coordinate with them in different meeting setup.  **Qn. Do you allocate budget for health sector?**  **-** The budget directly doesn't go to the health sector. We provide budget for the maintenance of the health post. If there is some equipment needed then, budget is allocated for them. Also, we are contributing in building birthing center. The budget is also allocated for the marginalized people, mother and child; there they are also under health. If we contribute to road construction, water purification, these also help in the health improvement. Therefore lot of indirect budget is allocated for the health sector. | In the case of health sector also we use planning process. We make them participate from pre planning process. In same way in periodic plan, district development plans we understand their emotion, needs, and programs and have discussion on them. In quarterly review and different meeting we discuss on how to carry the programs in related way and we move forward.  **Qn. Tell us about the budget planning in health sector?**  Local ministry segregates the budget for health sector. In the same way VDC also segregates budget in directed manner as per DDC. Every year we segregate some budget for health. In this way by separating budget, coordinating and by accumulating the health plan we are working side by side. | There is coordination in the entire program of the health sector. Though DDC directly don't work in the health program but DHO is its integral part. We are providing the helped needed to the health sector. Also, our VDC secretary is the chairperson of the HFoMC which shows linkage between DDC to health sector. We the coordination with the DHO is satisfactory.  **Qn. Do you allocate budget for health sector?**  -The budget for health sector is directly provided from the central level to DHO. If there is need of some help from the DDC sector. Then, we also give some input or budget to them. |
|  | **What sort of health related improvement have you observed in this area?** | There is only improvement seen in nutrition and child immunization. Another is, some improvement has been in field coordination. According to data, I have not seen but program are conduct continuous. Slightly improvements in immunization sector. Institutional delivery is also increased this year.  Nothing VDC are not declared as fully immunization. | Some are improvement in health sector such as family planning services, some improvement is vaccination.  Only one NGO are working in family planning program. We don't know about detail in health sector. | Lots of improvements were seen at Baitadi;  Institutional delivery has increased. People are aware. Nutritional status has improved. We have declared fully immunized district.  The people are aware about the personal hygiene, sanitation and hand washing. The children health is improving. | The improvement in the health sector is shown by the health indicators. There are many indicators which help us to determine the improvement met by the health sector. | On poush 12 we have declared 14 districts as fully immunized district. According to database I have experienced the situation has improved compared to past in birthing center, immunization.  **Qn. What is the present condition of nutrition and hygiene and sanitation in Bajhang?**  We have implemented multi-area nutrition program in 3 VDC. In area of hygiene and sanitation, after ODF declaration we have formed task force to make crucial plan. This task force after providing reports on basis of suggestion we will work.  **Qn. You said you announced multi-area nutrition program in three VDC. Which are the VDCs?**  The three areas are Kada; which is rural and occupy 54% area, Gadaraya and Channa VDC. | There has been improvement in health sector. Our children and mothers are out of risk of their untimely demise or sudden death. The local people are awareness about the health services which has increase the service utilization. Ultimately all these are improved the health condition of the people. |
|  | **What are the reasons behind this improvement?** | The reason behind the improvement, to help from district level health related focal person and other is line agency. Political support is the other factor that has helped to achieve these improvements and continuous monitoring has also helped in the improvement | The main reason is increasing in the awareness level of the people, service is good, FCHV to provide the information about the importance of immunization, nutrition, ANC checkup to community people.  Another reason is near different private hospital and governmental hospital has been working in this district so change in the behavior of the people. | There are different organizations in the district who are working to improve the health of the people. Also, the health worker is continuously working on it. | The main reason is increasing awareness in the people. Also many programs are also helping in this improvement. These are two things: software and hardware. The software part that is education, counseling is all enough. Now, we have focus in hardware part. Since there is still no operation facilities in Sindhuli district. Lot of indicators are low because there is no service provision in those areas. | Firstly all organization working in this field, DDC, DHO, VDC has given priority to this sector. NGO/INGO have played their role carefully. Second reason is that public awareness is increasing, educated population is increasing. In some way the agricultural production has increased which helps in increasing nutritional diet.  **Qn. what are the types of NGO and INGO working on health sector in Bajhang?**  Suhaara is conducting nutritional program. UNICEF is helping in hygiene and sanitation. Other organizations are also working. | It's due to education and awareness in the people. The Nepal government is also working on it; the government is committed towards it. |
|  | **What are the improvements in Maternal and Child health (MCH) sector?** | In maternal and child health sector, we watch the some improvement such as immunization, nutrition and child marriage has also improvement in this district. | Some VDC are declared in open defecation VDC but VDC name is don't know detail. It has some improvement in maternal and child health such as decrease in immunization, decrease in the severity of pneumonia, decrease in maternal and neonatal death etc. you can see indicator then already find out what type of improvement in maternal and child health. | Like I said, immunization is increased. The maternal deaths are low. People are aware about the personal hygiene. The children diseases are low. | **-** | It is in improvement status than before according to database. The maternal death rate has decreased as well as there is decrease in child death rate | The immunization is increased which has decreased the child mortality rate. The maternal deaths are low. From pregnancy to delivery, a woman has got help and care from the health worker which has improved their and their children's health. |
|  | **What can be done for additional improvement?** | Talking about additional improvement, in this Parsa District lack of human resources, lack of trained health worker, and lack of building. So these are fulfillment in the time district has been already improvement. | Regarding the additional improvement, first of all creating the awareness program in the community level then government provided to a lot of MNCH relation program implemented in respected district so we can some change saw the next year.  Sector. | Awareness program should be increased. | **-** | First of all number of birthing center should be increased. Trained manpower should be managed.  Sanction posts should be fulfilled. Where problems are being faced there should be facility of better road | To make improvements, the people working in the different sector should be dutiful and loyal towards their work and country. There must be management of medicines, manpower and other equipment needed in the health services. |
|  | **What are the problems you are facing in MCH sector?** | The main problem is facing in MNCH sector, lack of staff, lack of staff quarter, and lack of budget. Nepal government provide to staff but staff are not working properly because lack of staff quarter, Madhes revolution. So, staffs are transfer to other district. So these factors will be stop the central level then progress. | We have no new program in this district, lack of nursing staff, and lack of ambulance and also, NGOs are not working properly in this district.  Child marriage is also the problem | Our district is remote. The main problem in our district is accessibility. Due to the lack of communication and transportation, we are not able to provide the service. | Superstitious beliefs toward health. Staff transfer, lack of HR | Basically child death rate is increasing. Maternal death rate is increasing due to lack of awareness and being unable to provide the facility and services to the target group. There is problem of child marriage her.  presence of taboos like chhauapadi, child marriage, and superstitious beliefs about health | Our district is very remote. The main problem in our district is accessibility. Still today, there is no transportation or road way to many VDCs. Due to the lack of communication and transportation; we are not able to provide the service. Therefore, due to the lack of communication and transportation, we are facing problem in MCH sector.  Chhaupadi has been the problem. |
|  | **Do you know about District Case Investment (DIC) program?** | I don't know about DIC program. | I don't know about DIC program. | I don't know about that program. | I don't know about this program. | I have heard little information about DIC. it is basically based on what should be done in health sector, to make plans and manage budget for the program | I don't know about that program. |
|  | **What are the differences that you have noticed before and after implementation of DIC?** |  |  |  |  | For further development of health sector management is done and sources and materials for betterment of health sector is managed. We are able to make organization related to health sector responsible. |  |
|  | **Do you think that DIC should be continued to bring improvement in MCH sector? If yes, Why?** |  |  |  |  | Looking at its importance it should continue. I also hope that DIC can help in future for betterment of health sector.  **Qn. Why do you think DIC should continue?**  I think DIC should continue because on working by making plans can bring good results. |  |
|  | **Do you have any additional suggestions?** | Nepal government should be provided staff and their staff quarter, a lot of budget should be provide, a lot of trained health worker should be provide. And non-governmental organization such as IPS and UNICEF should be provided different type's health related program in time. | Government should be provide a lot of budget in this district. Budget is not giving in Madhes due to revolution is continued what happen; nothing there is lack of nursing staff and infrastructure is not well. Finally patient have go India (Raksaul) for operation and further checkup. All budget are relies to hill regions. | No, I do not have any. | The only thing is now; we have to focus in the hardware part. We have to build infrastructure and provide service in each and every cone. Only then, we will see increase in indicator. The software part is fully working and now again if we focus in the same thing we will get the stagnant result. Therefore, I think the I/NGOs should also focus in the increasing the service utilization. | NGO and nongovernmental organization are providing facility in efficient manner. Despite of that we are unable to provide services as per needs. So, to provide rights of people governmental office, nongovernmental organization should work genuinely to provide facility efficiently. | I want to say that, there are many NGOs and INGOs working in the country. They also tend to work in a geographically easy VDCs and districts where there is easy access of transportation and communication. Therefore, this should be stop. Since they are here to help poor and needy one, they have to work in places without access to road or communication too.  The Nepal government with its commitment to improve the health sector, maternal and child health; we from our side and the NGOs/INGOs should also support them to achieve it. |

**DIC Qualitative**

**Tool No. 2 Key Informant Interview (KII) Interview with Village Development Committee Secretary**

| **SN** | **Questions** | **Terai** | | **Hill** | | **Himalayan** | |
| --- | --- | --- | --- | --- | --- | --- | --- |
|  |  | **DIC** | **Non- DIC** | **DIC** | **Non- DIC** | **DIC** | **Non- DIC** |
|  |  | **Parsa** | **Sarlahi** | **Baitadi** | **Sindhuli** | **Bajhang** | **Darchula** |
|  | **How many years have you been working in this district?** | I have been working for 1 year in the Parsa district. | I have working only for 2 month in Bara District. | I am working as VDC secretary since six month. | I have been working here for 2.5 years. | I had completed 5 years as VDC secretary. | I have been working here for 5 years. |
|  | **What type of improvement has been currently made in planning process? Can you tell about 14 steps of planning process?** | Compare to previous year some are improvement in this year. First of all problem comes from Ward level. For example, women related program comes from women welfare commission, Dalit related problem come from Dalit sector, child related problem comes from child commission then planning process meeting is start then all agenda go through VDC council and big planning process comes from ilaka council and then district through 14 step are pass. | I am totally new VDC secretory. This is my first job. Just I am working in few months only. I am not involved in any planning process. So, I don't know about 14 Step planning. | 14 step planning starts from local level then it ensures to ward level. From there it proceeds to VDC level meeting. Then to VDC council and then passes to Illaka. After Illaka discussion meeting, we sit for integrated committee then we move to DDC meeting and then DDC council and then plans are submitted to upper level. | The planning process starts from the ward level. The plans that are raised in the ward level are passed in the VDC, then; those plans are passed from the planning process in the VDC.  **Qn. Can you tell me the whole planning process?**  -At first, the Planning is done in the ward level. The groups from different like agriculture, women, and children all have their own role. Then, after it is passed to the VDC level. Then we get the plans made by the wards.IN the planning process, there is participation of the entire member like: VDC secretary, Mother's group, local parties, local organization and member from NGO who works in the VDC level. We also prioritize the plans and then pass it to district level. | 14 step planning starts from local level then it ensures to ward level. From there it proceeds to VDC level meeting. Then to VDC council and then passes to Illaka. After Illaka discussion meeting, we sit for integrated committee then we move to DDC meeting and then DDC council and then plans are submitted to central level.  “Problems come from ward level like; women welfare committee and is taken to the ward level for discussion at VDC council”. | Due to country's situation, the work which should be done by the supreme authority is being by local people. We are working as the guideline provided by the higher level. We work from the planning process till its implementation. The main responsible person is the local people living there and the focal person of that place. They have to work on their area and have to make plans for their own place.  We must organize pre-planning workshop. After that, we make the village-wise planning where we include the target groups. We orientate the ward focal person and reach to every corner to know the demand of their area. Then, we collect the demand and make plans on the basis of this. After that, we organize an integrated planning meeting where we make an integrated plan focusing benefits to the community. After that, we again organize a VDC council where the local people, mother group, VDC stakeholder, Women awareness center, political parties are called for the council meeting. Then, the plans which were passed by the Ward integrated council are discussed in VDC council. Then, we prioritize the plans and make the final plan for the whole district. Then, they are passed from the VDC and we implement them. Our planning process in completed likewise. For implementation, we make one committee, at this part, the ward citizen forum is responsible for this. We, VDC secretary, Political parties, Health worker participate in the committee and introduce the approved plans. Then, together all implement the program. The plans are carried out by using local resources and personnel. Then, after implementation, we are responsible for monitoring of the programs. Whether the plans are being implemented or not are completely monitored by us. |
|  | **How do you coordinate in health sector?** | We are not providing budget but regarding the coordination in health sector especially for budget. First of all we are conducting planning meeting with VDC community people then create some agenda. This agenda pass from VDC level then make some plan and policy for budget then release and go for further work. We also coordinate with agriculture office, education, women welfare etc. | We are working in according to VDC. For budget, first of we conduct ward level meeting to different community people and broadly interaction to each and every person then go through according to minute. We are not giving budget only for giving FCHV incentive. We are providing only 500 hundred rupees per month to FCHV for incentive. Only we can provide 500 hundred for support to community people in health sector. | We coordinate with health facility. We support as per their needs.  **Qn. Please tell us about the budget secretion to health sector?**  We together discuss about the budget ceiling and develop the plan on basis of budget ceiling. After the discussion we select most essential need of the community. and necessary budget is provided to health facility for human resources, equipment’s, building construction etc.  Requiring big budget and human resource related support were transferred to national level and the low to medium budget activities were approved at district. | AS now, VDC secretary are the chairperson of the HFoMC, therefore we have main responsibility of health sector too. The review meetings also help us to know about the health section. The budget is also allocated for the mother and child from the VDC level. Not only that, we also allocated budget for medicines and other equipment needed for the health post. Likewise, there is close coordination with the health sector. | We coordinate with health facility and invite them in ward level meeting. We discuss in ward level meeting in presence of mothers group meeting about the support of VDC to health facility to improve the health status of new natal.  Q: Please tell us about the budget segration to health sector?  A: we discuss about the budget ceiling and develop the plan on basis of budget ceiling. Priority is given to need of the community and necessary budget is provided to health facility. Example supporting for human resources. | The budget is also allocated for the health sector. There is 10% budget for women, child and marginalized people. Importantly, we also make the plans covering the mother and child health. We also help in the health-related program such as, awareness program and for the physical infrastructures.  There are some problems in lack of ownership by the district as the plan are nationally driven**.** |
|  | **What sort of health related improvement have you observed in this area?** | Health is attached to every people and community. For example, Dalit community attached with health, women attached with health, child attached with health so every people are needed for health. And regarding the improvement in this area such as fully immunization has some improvement and it allocated some separate budget. Budget has separated For oxygen in the community people, and from VDC monthly incentive we can provide 500 hundred rupees per month to FCHV for help to community. So that's improvement we observed. | Some improvement then previous year. For example increasing in awareness and some budget. And also, Nepal government gave to budget. If no budget then nothing is improvement. | We have declared as fully immunized district similarly ODF. Decreased in maternal and child death. Increased ANC checkup and intuitional delivery. People know about the services of hospital, PHCC, HP. Increased user of family planning device.  Decreased malnutrition. | In the VDC, many organizations are working now. The activities such as: WASH related is being conducted. The health programs are also conducted time to time which has improved the health sector. | Awareness regarding health has increased.  People use nutritious food for diet.  Immunizing child. Increased institutional delivery.  Awareness about the timely checkup like BP  Decrease child death.  Seasonal disease has decreased. | Previously, there was no awareness program. The budget was not allocated for the health sector. If we make the plans for health sector, everybody was not convinced by it but due to the awareness now, many plans are directly or indirectly related to the health sector. Now, we have budget for women and child, high risk group and for marginalized population. The people are aware that the health is the main wealth of us. |
|  | **What are the reasons behind this improvement?** | The main reason is increasing in awareness and other is Budget. Another reason is effective counseling and communication by FCHV to community people. So, some improvement then previous year.  Access to communication, positive attitude of pregnant mother towards health service utilization and the support of the in-laws | The main reason is Sunaulo Hajar program start in Bar district for mother and child. So, some awareness creates to mother. | Support of different NGO/ INGO.  Increased awareness.  Proper coordination  Fulfilment of human resources.  Extension of birthing center. | The main reason behind these improvements is public awareness. Also the media is highly influencing in the health sector. There are contributions from the organization. These all are the reason behind improvements. | Increased awareness.  Support of different organization. Regular conducting of PHC/ ORC, EPI clinic  Access to health services. Another reason is suppprt from the family. | Firstly, the human resources (HP-incharge, ANM) is the reason for the improvement in the health sector. Since they are from the health line and they know better than us. They are working hard and counseling people for health check-ups. They provide information about the health relate event to the community people. |
|  | **What are the improvements in Maternal and Child health (MCH) sector?** | Regarding the maternal and child health, increasing in institutional delivery. No home delivery conducted, also some improvement is sanitation and hygiene maintain, nutrition, Immunization etc. all people are aware to know about child vaccination. That's improvement. | Talking about maternal and child health, some improvement then previous year. For example increasing in awareness, increasing in child vaccination, increasing in ANC checkup. Any type's disease suffering from child then mother take to child and go to private hospital, India and government hospital for further checkup. Another improvement reason is nutrition due to Sunaulo Hajar Program. | **Already mentioned in above question** | Yes, now the mothers are aware enough to go the health check-up during pregnancy. They also bring their children for health check-ups. If there is any health camp, then now we can see majority of the people are participating in that camps. | **Already mentioned** | The hand washing sector is improved. Previously, they even don't wash hand after defecation of before eating food. The people didn't know the disease due to unwashed hands. The HW provided the information about the importance of hand washing. And after long time, they started to know about the importance of hand washing and now they wash their hands. The HW also provided information about nutrition and condition during pregnancy and delivery. The daily diet and lifestyle of the women has also been improved. We also conducted awareness program in the remote villages too. |
|  | **What can be done for additional improvement?** | In my opinion, the main reasons behind the improvement people awareness are increasing. In the situation, all people and stakeholder are attending in small meeting. Another is communication. All people are take mobile phone so any information is share to fast.  DIC should be implemented for more years | In my opinion for additional improvement, more focus on target population and another is Nepal government should be provided a lot of budget for health sector. | Increasing monitoring.  Increasing community participation.  Broadcasting health massage from media. Strengthening mothers groups. Regularly conducting  PHC/ ORC. | **-** | Community participation should be given priority.  Increasing monitoring.  Providing awards for good performing staff and punishing not working.  Conducting training for mother groups.  Providing counseling to parents.  Conducting awareness program. Managing female staff for ANC and PNC checkup.  DIC should be implemented for more years | We should provide awareness to the people and along with that we must provide services to them. Only knowledge is not enough, there must be facilities together with it. For example, if we provide them knowledge of hand washing, then there must be water and soap to perform that activity by them. If we take about sanitation then, there must be suitable environment to take that action. |
|  | **What are the problems you are facing in MCH sector?** | The main problem is budget. In MNCH, we have no budget so we are very difficult to provide separate budget. Another is community people are not aware about MNCH so in my view government should be provide separate budget for pregnancy and lactating mother, FCHV incentive, for declaration of ODF and fully immunization sector. government should must be focus on target group, dalit, janjati etc. | The Main problem is road. In Bara district, most of the road is damage. Mothers are not going to hospital for pregnancy checkup, and child checkup. All children are not eating nutrition food due to lack of economic income. That's the main problem in Terai area.  Flood, appropriate infrastructure are also the problems | Lack of family support. Female of chepang community feeling shy to share their problems. Chepang women have less access to health services compare to other community. Chepang community lacks knowledge about sanitation and hygiene. | The problem is that we don't have a crowd village. There are small villages scattered all round and there lies jungle in the middle of the way which has hampered in service utilization. Also, my VDC doesn't have fertile land due to which we don’t have enough diet content. We need awareness in the nutrient portion. The villagers here need the knowledge about the nutrient content of the local foods. So that we can have further health improvements. Still there is no right environment for safe delivery in Sindhuli district. | Cultural superstitious belief is prevailing at bajhang.  Female are shying to share their feelings.  “There is lack of attraction of people towards services provided by government health facilities.  Proper water supply and health infrastructure. | There was very serious condition in previous year. There were no health facilities nearby and at time there were many maternal deaths. We even hear that the mother died on the way to hospital. Now, there are services in the villages itself, ORC clinic are ongoing in the ward which has improved the mother's health. |
|  | **Do you know about District Case Investment (DIC) program?** | I know about it. | No | I have not heard about it. | NO, I don't. | It works for MNCH.  It discuss about the  Responsibility of each stakeholder and encourage all to work. | I don't know. |
|  | **What are the differences that you have noticed before and after implementation of DIC?** | IC had successfully made all stakeholders accountable and supported to meet the health indicators considerably. |  |  |  | Full immunization district declaration.  ODF declaration district.  Decreased maternal death and child death. |  |
|  | **Do you think that DIC should be continued to bring improvement in MCH sector? If yes, Why?** | Yes, because all stakeholders come together for planning and budgeting for health. |  |  |  | It should be continuing. But timely monitoring and supervision is necessary. It encourages the entire stakeholder to perform their duty. DIC must include the entire field. It should not be limited to health only. |  |
|  | **Do you have any additional suggestions?** | For additional suggestion, government should be provided a lot of budget in Terai district for dalit, adibasi and janjai for education. In Terai, lack of road so Government should be managing road. That's problem. | For additional suggestion in my opinion, all people will be educated. If no education nothing is improvement. So all community people get education. Thank you. | No, I do not have any. | We need organization who works fairly for the people. There are many organizations working in the remote areas. I thank to them but still we need more who works for the welfare of the people. | Coordination of the entire field is necessary for betterment.  The entire stake holder should discuss about the priority of need. | This is 21^st^ century but still due to the geographical condition, the people still can't have the facilities they need. Therefore, Nepal government together with other organization should look more to the geographically difficult places. They must first analyze the living condition and the terrain of the places and bring program focusing on them. This would bring improvement in the heath sector. |

**DIC Qualitative**

**Tool No. 3 Focus group Discussion (FGD) with mother having 2 years old children and Female Community Health Volunteer (FCHV)**

| **S.N** | **Questions** | **Terai** | | **Hill** | | **Himalayan** | |
| --- | --- | --- | --- | --- | --- | --- | --- |
|  |  | **DIC** | **Non- DIC** | **DIC** | **Non- DIC** | **DIC** | **Non- DIC** |
|  |  | **Parsa**  **(P=8)** | **Sarlahi (P=8,FCHV=1)** | **Baitadi** | **Sindhuli**  **(P=7)** | **Bajhang** | **Darchula**  **(P=6,FCHV=1)** |
|  | **Where do you go to seek health services?** | **P1- P5** = we go in health post for health services.  **P6 – P7** = If the patient is serious, we go in Birgung National Medical Hospital  **P8** = If the patient is serious I go in India.  **Qn. For what services, do you to India for checkup?**  **P8=** For operation (Kidney stone)  **Qn.** **Do you not go Birgung National Medical College for operation?**  **P8**= In Birgung hospital, there is highly cost so I don't go. | **P1-** Kalaiya hospital(District Hospital)  **P2**= Kalaiya hospital and health post, private clinic  **P3**= Kalaiya hispital  **P4**= Kalaiya hospital and Birgung hospital  **P5**= Kalaiya hospital (District Hospital)  **P6**= Kalaiya hospital (District Hospital)  **P7**= Kalaiya hospital, private clinick  **P8**= Kalaiya hospital(District Hospital)  **P9**= Kalaiya hospital and Birgung hospital | **P1=** Health post  **P3**= Health post  **P7**= Hospital | **P6**= District hospital  **P7**=Everybody's home is nearby, therefore we come to district hospital | **P1=** Health facility, PHC ORC  **P5=** Group Health facility | **P1**= District hospital  **P2**-**P7**= District hospital. |
|  | **How far it is? How much time it takes to reach there?** | **P1 – P5** = only 15 minutes by walking  **P6 – P7** = It takes half an hour  **P8** = if go in India, 1 hour by Riksawa  **Qn. Any medical service available in your near house?**  **P1-P5**= small medical is near to houses.  **P6-P8 =** No medical is near houses  **Qn. Do you go first in traditional healer or to health post?**  **P1-P8 =** No go to traditional healer, we go to first health post, private hospital. | **P1=** 20 minute  **P2=**10 minute by walking  **P3=**15 minute  **P4=** If go In birgung, it takes half an hours by tempo  **P8-P9=** 10 minute  **Qn. Any medical service available in your near house?**  **P2=** Yes, the medical is near to house, only 5 minutes by walking.  **Qn. Do you go first in traditional healer or to health post?**  **P1-P9=** we go to first health post, private hospital and, Birgung hospital for checkup. No go to traditional healer, if the patient is not cure then go to traditional healer.  **Qn. How many Rupies take traditional healer for checkup?**  **P1=** only one hundred one rupies.  **Qn. How many Rupies take private clinic for checkup?**  **P2=**300 hundred checkup and self-brought medicine. | **P4=** it’s nearer. It takes five minute to reach.  **P3=** we can reach with in 15 minute.  **P6:** we take 5 minute to reach there.  **Qn. How far health post lies from the residential areas?**  **P6=** health post is nearer to residential areas.  **P7=** around one hour  **P2=** half an hour | **P1**= 15 min  **P2**= 5 min  **P3**= 15 min  **P4**= 10 min  **P5**= 10 min  **P6**= 15 min  **P7**= 15 min | **P2=** It takes 1 hour by walk and 15-20 minute by bus.  **P3=** around 1 hour  **P4=** 2 hours  **P5=** Half an hour  **Qn. How much time it takes to walkthrough the residential area?**  **P2=** it takes 2 days.  **P3=** 1 day  **P4=** 1 day  **Qn. How far health post lies from residential areas?**  **P1=** half an hour  **P2=** 1 day  **P3=** it is near to residential area  **P4=** 2½ or 3 hours  **P5=** 4 hours | **P1**-**P6**= 10-15 minutes  **P7**= 15 minutes by walking. |
|  | **What health benefits did you find?** | **P2=** All facility are good, they provide us the services is well.  **Qn. Are there services like family planning, delivery services, and OPD services?**  **P2=** Yes, there is all facility available in health post.  **Qn. In Birgung National Medical Hospital, what type of services is available?**  **P1-P8=** There is all facility are available such as x-ray, video x-ray, operation and all types of medicine.  **Qn. You, in India, is there like family planning, OPD, ANC services?**  **P8**= Yes, there is all facility, also different types of doctor available. | **P2=** In kalayia hospital, all services available such as ANC, PNC, Immunization, delivery services and OPD.  **P4=**There is no operation services, for operation we go to Birgung hospital  **P6=**There is no ambulance facility.  **Qn. Are there services like family planning and delivery services?**  **P8=**Yes, there is available  **P9=**Yes, there is available but medicine is not availbla only cetamol, cotrim, Zinc are available.  **Qn. In Birgung National Medical Hospital, what type of services is available?**  **P4=**There is all facility are available such as Pathilogy check, x-ray, video x-ray, operation, ambulance services and all types of medicine. | **P1=** ANC check up  **P2=** Child health care services  **P6=** whatever we have ask for we got it.  **P4=** I had gone to hospital for child health checkup  **P1=** family planning services  **P3=** for basic services we go to health post. For more services we go to district hospital. | **P2**= There are delivery services, ANC and PNC services.  **P6**= Operation is done here.  **P2**= immunization  **P1**= Plaster services  **P6**= Rs. 400 incentive is given for 4 ANC visit  **P2**=, X-ray, Video ray, blood checkifng | **P2=** Whatever we have asked for we got it.  **P3=** Family planning services. We don’t have stretcher now but it was there previously.  **P4=** Availability of stretcher  **P1-P5=** Some time there is no availability of medicine | **P2**= Immunization services, ANC check-up  **P4**= delivery services.  **P2**= there is operation service too.  **P7**= there were not operation service before but from last year, there is operation services.  The civir also comes for other services which are not present in the district hospital. |
|  | **How was the behavior of health workers?** | **P4=** Yes, there behavior, talking to patient is good.  **P8=** In India every doctor has good behavior.  **P6**= In Birgunj health post, staffs have given enough time to us for delivery time and also provide good counseling to us. | **P1- P4=** Yes, there behavior is good.  **P4=**In Birgung hospitals some staff habits are bad. I am Dalit (low caste, marginalized group) and am treated differently by the health workers. | **P1=** Theirs behavior is good.  **P7=** At district hospital some misbehave us.  **P6=** Health worker misbehave poor.  **P2=** Not only poor. Except the person who they Know, they misbehave all.  **P3=** Dr. Gunaraj Awasti is good. He behaves nicely. | **P4**= Yes, they give us good service. Their behavior is good  **P7**= They give us enough time. They provide good services. | **P2=** they behave properly  **P3=** they provide service properly  **Group=** they behave in well-mannered way. | **P1**-**P3**= Their behavior is good.  **P5**= We only go there when we need services. So, they behave nicely. |
|  | **Do you get medicines or not?** | **P8=** In health post, there are no enough of medicine, only Cetamol and ORS packe**t,** available there.  **P2**= My child is suffering from pneumonia and go in health post there is no medicine, doctor said to there is no medicine for pneumonia so but some medicine to be brought from other medical shop.  **Qn. which medicines do you has to buy?**  **P2=** I am not educated so I don't know.  **Qn. Is there the medicine available for whole year?**  **P2=** Not available, only some time available. | **P9=** In district hospital, there are no enough medicine  **P6=** District hospital only few medicines are provided but most of them take money for medicine.  **Qn. which medicines do you has to buy?**  **P7=** Only cetamol are providing too.  **Qn. Is there free medicine available for whole year?**  **P7=** we don't know about free medicine.  **Qn. Is there vaccine available for whole year?**  **P7- P9=** Yes, vaccine is available. | **P2=** We get cetamole only.  **P7=** No more medicine is available except cetamole  **P3=** we get iron tablet, albendazol is provided | **P7**= There are Iron Tablet, Zinc Tablet, cetamol etc.  **P2**= These are all free medicines. They give us ORS and all. We have to some medicines.  **Qn. which medicines do you have to buy?**  **P6**= If the medicines are available here, they provide us if not we have to buy medicines. | **P3=** Some time there is lack of medicine.  **Group=** the medicines mentioned by Nepal government we are get it in regular manner. | **P5**= Yes, there are medicines.  Iron tablets, cotrim, TB's medicines, Allergy creams, Join pain medicines etc.  **P7**= TB's medicines is free but all medicines are not available.  **P5**= There was shortages of medicines due to blockage  **P7**= And also due to flood last year. |
|  | **Was there appropriate place for health check-ups?** | **P1 –P5 =** There is separate room for checkup.  **P7=** Available in separate room  **P8 =** we go to hospital for ANC checkup, there are separate room | **P1-P9=** Yes, there is a lot of room in district hospital and separate room for checkup.  **P3=** In health post, only one room for women so call us one by one. | **P7, P4 and P1=** they keep us in separate room.  **P3=** confidentiality is well maintain.  Your health facility consist of only two rooms, how do they maintain confidentiality?  **P1=** except the patient every one wait outside the room.  **P6=** nursing staff checkup us maintaining privacy. | **P2**= There are separate room.  **P1**- **P7**= Yes, there are separate room. | **P3 - P5=** They keep us in separate room and discussion is done based on subject matter.  **Group=** there is separate room and confidentiality is also maintained well.  **P3=** there is separate room for boy and girl and there is separate room for pregnant women for checkup and delivery. | **P5**= There is separate room.  **P6**= There is one room for women. They call us one by one.  **P7**= there is number system. They go one by one after calling number |
|  | **Do you know about District Investment case (DIC) program?** | **P1-P8=** No, we don't know | **P1-P9=** we don't know | **Group=** nope we have not heard about it. | **P1**-**P7**= No we haven't heard about this. | **Group=** No. we had heard about it just yesterday. | **P1**-**P7**= No, we don't know. |
|  | **What are the differences that you have noticed before and after implementation of DIC?** | Don’t know about DIC | Don’t know about DIC | Don’t know about DIC | Don’t know about DIC | **P2-P5 =** Yes, there is difference.  **P5=** They didn’t use to come for ANC PNC checkup, now they are coming. Institutional delivery has increased. The mother’s group meeting is now held in regular manner.  **P2-P5=** Now the health workers also participate in mother’s group meeting.  **P5=** When people except for us, if talk to community people they give interest because we are known to them and they think we repeat same thing always. When new people tell them about something they follow it.  **P2=** Previously it used to be closed on Saturday. Now the services are provided in regular manner. Previously many people had to return back since the health post used to be closed on Saturday.  **P4=** There is awareness about safety of institutional delivery and institutional delivery has also increased.  **P5=** *“previously the community people did not have faith in health workers but now they visit health facility for receiving the incentives and services and the health workers are also attending the Health mothers group meeting”.*  **P1-P3=** There is increase in institutional delivery due to well manner of ANM | Don’t know about DIC |
|  | **What programs running under Maternal and Child health (MCH)?** | **P2-P5=** Sunaulo Hajar  **P4=** provide nutritious food to pregnant and lactating mother  **P7=** Balvita provide health post for child suffering from malnutrition for nutritious diet.  **Qn. Is there any MNCH related program?**  **P8=** Only we know about Sunaulo Hajar and Balvita program.we do not know detail. | **P2=** Sunaulo hajar din, they must provide to information about ANC, PNC, child vaccination for community people.  **P5=** Another program is conduct balvita for child. If the child are underweight then go in hospital, health worker are given balvita for nutritious food.  **Qn. You are involving in mother group meeting?**  **P9=** There is regular conducted mother group meeting. In mother group meeting we are all present and discuss about different topic such as nutrition, ANC, Family planning and breast feeding etc.  **Qn. Is there any MNCH related program?**  **P3-P6=** only Sunaulo Hajar din and another is don't know. | **P1=** Suaahara is working here.  **P4=** No one is working for us  **P7=** We have mothers group  **P2=** Suaahara is working for nutritional status. | **P1**=We worked hard in the community to aware golden 1000 days’ mothers for service uptake. They are more aware than before. They are also more proactive in seeking information  **P3**= They provide us bed nets.  **P7**= We get Rs. 1400 if we have done 4 ANC visit and institutional delivery.  **Qn. Are there any other programs?**  **P2**= No, this is it.  P1=We worked hard in the community to aware golden 1000 days’ mothers for service uptake. They are more aware than before. They are also more proactive in seeking information | **P1=** neonatal program from JSI  **P3=** nutrition program  **P4=** Ama surachha program | **P3**= There is Suaahara program for mothers.  **P7**= They say, the Suaahara program is also finished.  **P4**= no, I have seen its employee here.  **P7**= Yes, it is going to be phase out. Half of its staff is cut-off.  **P2**= Suaahara has given us chickens, seeds for kitchen garden.  They have also give us information about pregnancy care, delivery care and neonatal care.  **P7**= They have given 5 chicken and also had come to monitoring.  **P5**= We also get delivery and transportation incentives, Clothes to mother and child.  **P7**= Yes. It has been 2 years that the mothers are getting clothes. It is called Nyano-jhola. |
|  | **What programs should be conducted to improve the health condition of mother and child?** | **P1-P8=** we do not know. | **P9**= we should have different types of services such as FCHV training, a lot of program and their incentive, a lot of medicine available in health post etc.  **P2=** we have separate women's doctor, ambulance service for pregnancy women. The main problem is Road. Government should have made road. | **P5=** Many village women are still not aware about the hygiene and sanitation.  **P7=** Suaahara should be continued  **P2=** Fulfilling sanction post.  **P1=** Timely referring of severe case. | **P7**= There is no blood bank service in this district. We first need blood bank service. Then, we also need ICU services too.  **P2**= The doctor are not on time. We have to wait them for hours. What I want is when we are here for check-up there must be doctor available anytime. | **P1=** Awareness program  **P4=** Vocational skill enhancing program to improve their status.  **P3=** Human resources should be increased. | **P5**= There must be program related to nutritious food to pregnant and lactating mothers.  **P7**=They must provide information and training to the pregnant and mothers about nutritious food. There must be awareness program related tp maternal health and its problem.  **Qn. Any other?**  **P7=** The program related to sanitation should also be conducted. This will also help mothers for their good health.  Previously, the villagers didn't even plant spinach. Suaahara has taught us about its importance and now we plant green vegetables in our garden too.  **P5**= they have started from the base of good health I,e form handwashing. They have teach us about handwashing time, before and after eating, after defecation, before feeding child.  **P7**= still there is problem of conducting mother's group meeting. The poor family have to work on daily wages so they son't have time to attend meeting. Their income in on daily labor. There is no employment opportunities.  **P5**= they go to India and work there on daily wages.  **P7**= There is no agriculture area here therefore they have to do labor for income. Also, there is no refreshment in mother's group meeting.  There is problem of water also. Previously we used to drink water of Mahaklai river but due to rapid population growth, sewage mixing snd open defecation, it worth nothing. This should also be maintained. |
|  | **Are you included during planning process in VDC?** | P1-P8= we are not called; we are not include in VDC planning process.  P7: No, I don’t know about this annual planning. We are not consulted during the process. Leaders do that themselves | **P1-P9=** we are not included in any VDC planning process. | **Group=** we have not participated. | **P2**= No, we are not involve in the planning process.  **P6**= We don't know about this. | **Group=** we have not participated.  **P2=** one representative from FCHV participate there. | **P7**= They don't call us.  **P5**= They used to call us in planning process but while allocating budget they do it by themselves.  **P7**= previously they used to give us incentives for dress, rs. 100 per month. But now this place has become municipality and now there is no money. They say its municipality, we don't provide money.  **P5**= I lobby to make road from municipalities to upper area. It been 2 years but they say there isn't money for this time.  The politicians make plans, they play main role in it and they allocate budget into themselves. |
|  | **Do you have any additional suggestions?** | P2= There is must be a lot og program, must be medicine facility. We are labour and farmer, we don't know about detail and don’t have enough money.  P4- “A child from an affluent family even after spending around five hundred thousand Nepali Rupees and they could not save the child, died of a minor disease like diarrhea, it’s so sad!”. So should be provision of treatment and money for poor. | **P2=** The main problem is road in Terai. So, government more focuos on made road, also electricity is must be necessary, so electricity government should be provided.  **P5=** For children and pregnancy women, Nepal government should be provided nutritious food for dalit and janjati community. | **P1=** Suahara should be continue.  **P7=** thanking for your visit.  **P3=** we have the problem of drinking water. Due to lack of water many people did not use toilet. | **P7=** The hospital area must be clean. | **P4=** Thank you for visiting us. | **P7**= If there is any support from your side, then please remember Darchula district.  **P5**= Many people come and go; they only talk big thinks. If you can do anything then support here.  **P7**= We have to go to india for everything. From salt to small things to clothes, and even for medicines, we have to go there.  The Khalanga HP is build big and is equipped but there is no village near to it. Even HW doesn't stay there. Therefore, lot of resources has been lost there. If that money was utilized for some other thing then, it would have given better result. |
