# Supplementary material for: Investment case approach for equitable access to maternal neonatal and child health services: Stakeholders’ perspective in Nepal
Source: PLoS One. 2021 Oct 5;16(10):e0255231. doi: 10.1371/journal.pone.0255231 (PMC8491871; doi:10.1371/journal.pone.0255231)
Supplement: Attachment — Submitted filename: Review2.docx [file pone.0255231.s002.docx]

Thanks for giving me the opportunity to review the paper. Please see my comments as below.

Study setting: The study was conducted in in June 2014 in purposively selected

districts based on HDI values, three intervention and three comparison districts. Delete one “in” in the first sentence.

In the method section: Please add a brief paragraph on data collection specifying: Who were the FGD facilitators (what were their qualifications, what training did they receive in FGD facilitation)? Was there a note-taker? Where did the interviews take place? What was the consent process? Were the information collected in of KIIs and FGDs different?

In the analysis section, please specify the following: How was the coding done on full transcripts? How many people were involved in this? How was the iteration done? Was any double-coding done to check inter-coder reliability? How have you checked the interpretation validity and descriptive validity?

Did you combine findings from KIIs and FGDs while presenting the results?

In the results section, please present some background info of the participants. After each quote please write some info of the participants: age, sex, years of experience, designation, intervention or comparison group.

Discussion:

In general, discussion needs to be better situated in the literature. Look for other studies of interventions or program/policies that might have worked to address your specific issues, even from other countries.

What are the limitations of the study? Please describe in the text.

Conclusion: “It was seen that districts without IC showed numerous flaws on implementation”. What would be your recommendation for them?

Finally, the manuscript needs editing by a native English speaker. There are many inconsistencies in grammar throughout the manuscript.
