# Supplementary material for: Investment case approach for equitable access to maternal neonatal and child health services: Stakeholders’ perspective in Nepal
Source: PLoS One. 2021 Oct 5;16(10):e0255231. doi: 10.1371/journal.pone.0255231 (PMC8491871; doi:10.1371/journal.pone.0255231)
Supplement: Attachment — Submitted filename: Response to Reviewers.docx [file pone.0255231.s004.docx]

**Dr. Bidhubhusan Mahapatra, Ph.D.,	15 May 2021**

**Academic Editor**

**PLOS ONE**

Re: Comments addressed for the manuscript " **Investment Case Approach for Equitable Access to Maternal Neonatal and Child Health Services: Stakeholders’ Perspective in Nepal** "

**Dear Dr. Bidhubhusan**,

Thank you very much for providing opportunity to revise our manuscript. We have attempted to address all the comments in our revised manuscript. Please find the responses to each comment below;

1. Please amend your list of authors on the manuscript to ensure that each author is linked to an affiliation

**Response:** Thank you for your valuable comment. We have addressed it in page number 33 (Line no: 799 to 813) as:

**Authors Contributions**

**Conceptualization:** Janak Kumar Thapa, Maria Delius and Doris Stöckl.

**Data curation**: Raj Kumar Sangroula and Janak Kumar Thapa and Dip Narayan Thakur.

**Formal analysis**: Janak Kumar Thapa, Raj Kumar Sangroula.

**Funding acquisition**: The authors received no specific funding for this work.

**Investigation**: Janak Kumar Thapa and Maria Delius and Doris Stöckl.

**Methodology**: Janak Kumar Thapa, Maria Delius, Doris Stöckl, Asha Pun, Meena Thapa, Raj Kumar Sangroula, Mahesh Kumar Maskey, and Dip Narayan Thakur.

**Project administration**: Janak Kumar Thapa, Doris Stöckl and Maria Delious.

**Software**: Janak Kumar Thapa and Raj Kumar Sangroula.

**Supervision**: Janak Kumar Thapa and Maria Delius and Doris Stöckl

**Visualization**: Asha Pun, Meena Thapa and Raj Kumar Sangroula.

**Writing** – original draft: Janak Kumar Thapa.

**Writing – review & editing**: Maria Delius, Doris Stöckl, Asha Pun, Raj Kumar Sangroula, Mahesh Kumar Maskey.

2. Your ethics statement should only appear in the Methods section of your manuscript. If your ethics statement is written in any section besides the Methods, please move it to the Methods section and delete it from any other section. Please ensure that your ethics statement is included in your manuscript, as the ethics statement entered into the online submission form will not be published alongside your manuscript.

**Response:** Thank you for your valuable comment. We have addressed it in page number 10 (line number 244 to 249) as:

**Ethical considerations**

The ethical approval was obtained from Ethical Review Board (ERB) of Nepal Health Research Council (reference no. 1296) and the Centre for International Health LMU (CIH^LMU^). Written informed consent was obtained from the participants before interview. The principal researcher has securely stored the signed informed consent forms.

3. Thank you for stating the following financial disclosure:

"NO - Include this sentence at the end of your statement: The funders had no role in study design, data collection and analysis, decision to publish, or preparation of the manuscript."

At this time, please address the following queries:

Please clarify the sources of funding (financial or material support) for your study. List the grants or organizations that supported your study, including funding received from your institution.
State what role the funders took in the study. If the funders had no role in your study, please state: “The funders had no role in study design, data collection and analysis, decision to publish, or preparation of the manuscript.” If any authors received a salary from any of your funders, please state which authors and which funders. If you did not receive any funding for this study, please state: “The authors received no specific funding for this work.”

**Response:** Thank you for your comment. We have addressed it as, “The authors received no specific funding for this work” in page number 33 (line number 796 to 797)

4. Can you please upload an additional copy of your revised manuscript that does not contain any tracked changes or highlighting as your main article file.  This will be used in the production process if your manuscript is accepted.  Please amend the file type for the file showing your changes to Revised Manuscript w/tracked changes.

**Response**: Thank you. We have uploaded both track change version as well as final version without track changes.

**Response to Reviewer 1**

Reviewer #1: It is an interesting piece to read and authors have tried to cover all the aspects of the DIC program. However, it feels the introduction section can be more tightly written, to give the reader and idea of what to expect in the paper in first 2 paragraphs. Thorough English editing is needed, and too much citation of past work in the data section instead of the original work done by the authors. Few detailed observations are given below-

**Response:** Thank You for your valuable comments which mean a lot for us. We have edited the introduction section and rewritten the portion. Page 3,4 and 5 (line number 51 to 111)

**Comment: The first line in introduction is about MDGs, isn’t it’s too late to talk about MDG goals? Why not state and compare where Nepal is as per SDG goals which is reducing it to less than 70 per hundred thousand live births.**

**Response:** Thank you for your comment. We have edited the sentences as, “Nepal has achieved several Millennium Development Goals (MDGs) and has been close to attaining the target of reducing maternal mortality by 75% from 258 to 213 per 100,000 live births (1) and is progressing towards achieving the SDG target of 70/100,000 live birth (2)” – page number 3 (line number 51 to 54)

**Comment:** On page 3 the sentence says “In this regard, it is important to have actions to achieve higher quality at local level……” author needs to elaborate a little higher quality in what?
Provide reference for the sentence “The knowledge of evidence-based approaches and effective frameworks for maternal health management is low among local stakeholders.”
Better to provide examples on who all can be stakeholders in the introduction itself..

**Response:** Thank you for your comments. We have edited the introduction portion. page number 3, 4 and 5 (line number 51 to 111)

We have also mentioned the local stakeholders in the introduction portion as The main actors for implementation of the DIC programs were District Health Officers, Public Health Nurses, Local Development Officers, VDC secretary, frontline Health workers from the community level, FCHVs and so on –page number 4 (Line number 91 to 93)

**Comment:** In study participants- describe who is program implementers. And role of each key stakeholders in the ongoing DIC program.

**Response:** Thank you for your comments. We have re-written the study participants in the methods section. – page number 6 and 7 (line number 145 to 164)

**Comments:** Data collections section needs to be rewritten with more clarity and details like saying only interviews is not enough, authors should mention whether its face to face interviews, who is conducting interview? Whether authors themselves did it? Who did the translation, validity of translation? Who all was involved in the analysis?

**Response:** Thank you for your valuable comments. We have re-written the data collection section in method part of the manuscript. – page number 8, 9 and 10 (line number 177 to 243)

**Comment:** On page 11 what does KII means? Need to give the full form.

**Response:** Thank you. we have mentioned the full form of KII as Key Informant Interview in the manuscript. Page number 6 (line number 146)

**Comment:** What was the division of sample between intervention and control districts? Was it same or different?

**Response:** The sample size both the intervention and comparison districts were same and level of the study participants were also same. We have included it in the methods section. - page number 5 (line number 113 to 121)

**Comment:** Data section part is very poorly written, most of the sentences are taken from some other studies instead of writing what was done for the present study. It will be better to note down all the steps for clarity of the readers in case someone wants to replicate this approach in future. For example- on page 12 instead of writing that “researchers’ interpretations were controlled and cross-checked….” Please elaborate what was done to control and cross-check?

**Response:** Thank you. We have re-written the part. – page number 8, 9 and 10 (177 to 243)

**Comment:** On page 12 it is mentioned “adopted an analytical framework of structure of perception and experiences……….” Which framework is this? No details are given about this in the paper.

**Response:** Thank you. we have omitted the sentence.

**Comment:** In second paragraph of the discussion authors have written “knowledge across nations through media or staff transfer….” Shouldn’t it be knowledge across districts?

**Response:** Thank you. We have edited the sentence. – page number 20 (line number 493 to 494)

**Comments:** On page 22, “Females, marginalized people and disabled people were also found to be involved, however this is not confirmed by literature”. Why this needs to be confirmed by literature? If this is a new program, and the present study is an evaluation authors should state their findings with confidence without worrying about literature. Moreover, this sentence feels contradictory to what is mentioned in the results- “women in both study groups, that they or their family members are often not consulted during the village level annual MNCH planning”. Looks like there is discrepancy in what women are saying and what other stakeholders are saying regarding involvement of village population. A focus on this should be added in the discussion.

**Response:** Thank you. We have edited the discussion part as commented and we have added some literatures in the discussion part as well. - page number 21 (line number 505 to 514)

**Responses to reviewer- 2**

**Comment: Study setting: The study was conducted in in June 2014 in purposively selected**

**districts based on HDI values, three intervention and three comparison districts. Delete one “in” in the first sentence.**

Response: Thank you for your valuable comment. we have rearranged the sentence- page number 5 (line number 113 to 114)

**Comment: In the method section: Please add a brief paragraph on data collection specifying: Who were the FGD facilitators (what were their qualifications, what training did they receive in FGD facilitation)? Was there a note-taker? Where did the interviews take place? What was the consent process? Were the information collected in of KIIs and FGDs different?**

Response: Thank you for your valuable comment. For the collection of data, 3 FGD facilitators with Bachelor’s in Public Health degree with 2 years’ experience and three note takers with Bachelor’s degree in Public Health with 2 years’ experience in qualitative research were recruited. The same teams were involved in taking Key-informant interviews too. Three days’ intensive qualitative research-related training was given to the team by the lead researchers and other co-researchers who were expert in qualitative research. The training consisted of a different session on qualitative research including an introduction to qualitative research, scope, approaches, methods, orientation to the tools, discussion, mock session and exercise in real field setting. The key-informant interviews took place in the District Health Offices, Local Development Offices, Health Posts, Districts Hospitals of the respective districts. The themes of the information collected from KIIs and FGDs were same. Both included the impact of Investment Case approach on maternal and child health status. – page number 8 and 9 (line number 199 to 211)

**Comment: In the analysis section, please specify the following: How was the coding done on full transcripts? How many people were involved in this? How was the iteration done? Was any double-coding done to check inter-coder reliability? How have you checked the interpretation validity and descriptive validity?**

Response: Thank you for your valuable comment. In the “analysis of the data” and Rigor, Reliability, and Validity part of the method section, we have edited the part as: We highlighted various phrases in different colors corresponding to different codes in which each code described the idea or feeling expressed in that part of the text. – page number 9 (line number 219 to 221)

For coding and making themes from the codes, the lead researcher was mainly involved. The co-researchers assisted in the process. For all the steps of data analysis, the lead researcher was mostly involved and in discussion with the co-researchers, the analysis was finalized. All the co-authors agreed- following the discussion while finalizing the codes and the themes. – page number 9 and 10 (line number 225 to 230)

Study tools were finalized in consultation with the qualitative as well as subject experts, and the data were collected by well-trained field researchers. – page number 10 (line number 233 to 234)

The collected data were checked by the lead researcher and other co-researchers for their accuracy and quality for ensuring descriptive and interpretation validity. In case of missing data, the participants were followed up on the next day. – page number 10 (line number 238 to 241)

**Did you combine findings from KIIs and FGDs while presenting the results?**

Response: Thank you for your valuable comment. we have combined the finding from KIIs and FGDs while presenting the results.

**In the results section, please present some background info of the participants. After each quote please write some info of the participants: age, sex, years of experience, designation, intervention or comparison group.**

Response: Thank you for your valuable comment. We have revised the sections as per the comments given.

**Discussion:**

**Comments: In general, discussion needs to be better situated in the literature. Look for other studies of interventions or program/policies that might have worked to address your specific issues, even from other countries.**

Response: Thank you for your valuable comments. We have added related literatures for discussion. They appear as:

A study conducted for Evaluation of Accelerating the Implementation of the Investment Case for Maternal, Newborn and Child Health in Asia and the Pacific Programme concluded that IC approach brought about positive changes and the indicators are showing upward trends - page number 20 and 21 (line number 502 to 505)

The IC approach solely focuses on maternal, neonatal, child health and nutrition. From an IC evaluation study, the district health managers recommended for comprehensive planning approach which would cover other health areas too- page number 22 (line number 529 to 532)

A project named “The East Africa Maternal Newborn Child Health project (EAMNeCH)” was implemented in four vulnerable countries of Africa which focused on quality, demand, supply of maternal, neonatal and child health services and favorable policy environment. The results of the project contributed toward also improvement in MNCH indicators - page number 22 (line number 547 to 551)

According to a systematic review conducted in low income countries in Africa also supported the findings in which lack of skilled health services providers and access to health facilities were the barriers to utilization of MNCH services - page number 22 and 23 (line number 553 to 556)

This was also supported by a case study conducted in Nepal. There was increase in institutional delivery by establishing birthing centers near to the community - page number 23 (line number 566 to 568)

Case studies from Mozambique, Nepal and Rwanda have shown that improved health financing, decentralization of decision making and service delivery, task-shifting, development of partnerships, adequate Community Health Workers and community engagement were the important drivers for uplifting maternal and child health - page number 25 (line number 612 to 616)

**Comment: What are the limitations of the study? Please describe in the text.**

The study has some limitation: the study was only conducted in 6 districts (3 intervention districts and 3 control districts) out of 21 intervention and 56 comparison districts. - Page number 25 (line number 617 to 618)

**Conclusion: “It was seen that districts without IC showed numerous flaws on implementation”. What would be your recommendation for them?**

Response: We have added the part as “It is also recommended to implement similar or such type of approach in other non-intervention districts with strong supervision and monitoring in order to strengthen planning and budgeting in MNCH related programs. – page number 26 (line number 636 to 639)

**Comment: Finally, the manuscript needs editing by a native English speaker. There are many inconsistencies in grammar throughout the manuscript.**

Response: Thank you for your valuable comment. The manuscript has been edited by a native English speaker.

With Best Regards

Janak Kumar Thapa (Corresponding Author), PONE-D-20-40391R1
Investment Case Approach for Equitable Access to Maternal, Neonatal and Child Health Services: Stakeholders’ Perspective in Nepal.
