# Supplementary material for: Investment case approach for equitable access to maternal neonatal and child health services: Stakeholders’ perspective in Nepal
Source: PLoS One. 2021 Oct 5;16(10):e0255231. doi: 10.1371/journal.pone.0255231 (PMC8491871; doi:10.1371/journal.pone.0255231)
Supplement: Attachment — Submitted filename: Response to reviewers.docx [file pone.0255231.s005.docx]

**Bidhubhusan Mahapatra, Ph.D.	8 July, 2021
Academic Editor
PLOS ONE**

Re: Comments addressed for the manuscript " **Investment Case Approach for Equitable Access to Maternal Neonatal and Child Health Services: Stakeholders’ Perspective in Nepal** "

Dear Editor,

Thank you very much for providing opportunity to revise our manuscript. We have attempted to address all the comments in our revised manuscript. Please find the responses to each comment below;

**Comments:** We note that your author list was updated during the revision process. Please complete our Authorship Change Form by following this link: <http://journals.plos.org/plosone/s/file?id=13d0/plos-one-change-to-authorship-form.docx> If you are adding or removing more than 2 authors, you can complete multiple forms and submit as many forms as needed to reflect the updates. Please return the form(s) as an attachment by emailing [plosone@plos.org](mailto:plosone@plos.org) or by uploading it as a submission file labeled with the file type ‘Other’. Please note that if your manuscript is accepted, we will not be able to complete the publication process without the completed form.

**Response**: Thank you for your valuable comment. We have completed the Authorship Change Form and uploaded it.

We also have updated out acknowledgement section as: We would like to acknowledge the support received from UNICEF Nepal, Little Buddha College of Health Science, Mr. Dip Narayan Thakur, Mr. Raj Kumar Subedi, Ms. Monica Manandhar, and Ms. Radhika Sapkota for their support during the study. (page number 28; line number 614-616)

**Comment:** Please ensure that you refer to Table 2 in your text as, if accepted, production will need this reference to link the reader to the Table.

**Response**: Thank you for your comment. We have referred to table 2 in the text (page number 10; line number 211).

With Best Regards

Janak Kumar Thapa (Corresponding Author), [**PONE-D-20-40391R3**]
Investment Case Approach for Equitable Access to Maternal, Neonatal and Child Health Services: Stakeholders’ Perspective in Nepal.
